# Genomic and Phenotypic Characterization of a Wild Medaka Population: Towards the Establishment of an Isogenic Population Genetic Resource in Fish

**DOI:** 10.1534/g3.113.008722

**Published:** 2014-01-09

**Authors:** Mikhail Spivakov, Thomas O. Auer, Ravindra Peravali, Ian Dunham, Dirk Dolle, Asao Fujiyama, Atsushi Toyoda, Tomoyuki Aizu, Yohei Minakuchi, Felix Loosli, Kiyoshi Naruse, Ewan Birney, Joachim Wittbrodt

**Affiliations:** * European Bioinformatics Institute, Wellcome Trust Genome Campus, Hinxton, Cambridgeshire, UK; † Centre for Organismal Studies, Heidelberg University, Germany; ‡ Karslruhe Institute of Technology, Karlsruhe, Germany; § Comparative Genomics Laboratory, Center for Information Biology, National Institute of Genetics, Shizuoka, Japan; ** National Institute for Basic Biology, Laboratory of Bioresources, Okazaki, Japan

**Keywords:** Medaka, inbreeding, population genomics, strain specific features

## Abstract

*Oryzias latipes* (medaka) has been established as a vertebrate genetic model for more than a century and recently has been rediscovered outside its native Japan. The power of new sequencing methods now makes it possible to reinvigorate medaka genetics, in particular by establishing a near-isogenic panel derived from a single wild population. Here we characterize the genomes of wild medaka catches obtained from a single Southern Japanese population in Kiyosu as a precursor for the establishment of a near-isogenic panel of wild lines. The population is free of significant detrimental population structure and has advantageous linkage disequilibrium properties suitable for the establishment of the proposed panel. Analysis of morphometric traits in five representative inbred strains suggests phenotypic mapping will be feasible in the panel. In addition, high-throughput genome sequencing of these medaka strains confirms their evolutionary relationships on lines of geographic separation and provides further evidence that there has been little significant interbreeding between the Southern and Northern medaka population since the Southern/Northern population split. The sequence data suggest that the Southern Japanese medaka existed as a larger older population that went through a relatively recent bottleneck approximately 10,000 years ago. In addition, we detect patterns of recent positive selection in the Southern population. These data indicate that the genetic structure of the Kiyosu medaka samples is suitable for the establishment of a vertebrate near-isogenic panel and therefore inbreeding of 200 lines based on this population has commenced. Progress of this project can be tracked at http://www.ebi.ac.uk/birney-srv/medaka-ref-panel.

Defined genetic reference panels of inbred lines with divergent genotypes often have been exploited in genetics (reviewed in Flint and Mackay 2009). Broadly there are two approaches to creating such panels. The first involves crossing a small number of genetically distinct founders, followed by a number of interbreeding steps leading to an outcrossed population (Bailey 1971). The outcrossed population is then inbred again to form recombinant inbred lines. The second approach involves capturing wild individuals from a population and inbreeding them to give near-isogenic wild lines. Both methods result in a panel of inbred lines that has specific benefits for genetic mapping compared with the use of outbred individuals. First, as the same genotype can be produced multiple times from the panel, genotypic and phenotypic resources can be shared on the same panel between research groups and experiments. Second, the ability to make measurements from different individuals of the same genotype can overcome much of the nongenotypic study variance. Finally, the environment can be systematically varied between many individuals of the same genotype, allowing dissection of interactions between genotype and environment, something that is not achievable in standard outbred populations. Recombinant inbred lines provide a more straightforward route to full characterization of the complete genetic components of a population, often relating phenotypes back to the founder strains. However, mapping resolution is restricted by the low number of recombinations available in the panel, and the overall diversity of the panel is limited by the diversity of the input founders, which can limit analysis of interesting traits. In contrast, near-isogenic wild lines usually have both greater diversity of alleles (ideally with a consequent diversity in phenotypes) and have far better recombination patterns, allowing for the discovery of more genetic influences and finer mapping of these loci. With the falling price of sequencing, determining the complete genotype for each line is no longer so onerous, leaving the inbreeding process itself and obtaining sufficient distinct lines to overcome multiple testing issues as the two major limitations of a near-isogenic panel.

Recombinant inbred lines or near-isogenic lines have been developed in many different species during the last 50 years. For example, in the maize genetics community the set of 302 IBM maize strains has been a cornerstone of both basic and applied research (Sharopova *et al.* 2002; Fu *et al.* 2006). In the *Arabidopsis* community, the collection of 107 different wild accessions has allowed the exploration of the genetic determinants of a number of phenotypes and their relationship to the environment (Atwell *et al.* 2010). The development in *Drosophila* of both recombinant inbred lines (King *et al.* 2012) (>1700 lines) and near-isogenic wild lines (Mackay *et al.* 2012) allows the genetic dissection of phenotypes coupled with the excellent transgenic and other resources in this organism. The yeast research community have used crosses between wild and laboratory strains (Bloom *et al.* 2013), or surveys of wild species in related yeasts (Liti *et al.* 2009) to explore genotype to phenotype associations. In vertebrates, the emphasis has been more on recombinant inbred lines. These include the BNxSHR cross in rats (Pravenec *et al.* 1989) and the Black6/DBA cross in mouse (Peirce *et al.* 2004), both of which lead to a number of interesting traits being mapped in these species. The Mouse Collaborative Cross is the largest recombinant inbred line experiment undertaken in vertebrates (Collaborative Cross Consortium 2012) and is already showing promising results, although the mapping resolution will remain in the megabase range. So far the long generation times and difficulty in laboratory husbandry of wild individuals has prevented, to our knowledge, the establishment of a near-isogenic panel from the wild in any vertebrate species.

During the last decade, the model vertebrate medaka (*Oryzias latipes*) has been rediscovered beyond Japan for its developmental genetics, genomics, and evolutionary biology (Wittbrodt *et al.* 2002; Takeda and Shimada 2010). The physiology, embryology, and genetics of medaka have been extensively studied for the past 100 years. The long history of medaka research and its amenability to inbreeding make this species very well suited for genetic studies and especially for establishing a reference panel of inbred lines. A large number of wild catches have been collected to establish laboratory strains and highly inbred lines, which form a unique repository for genomic and population genetic studies. Because of this and the easily accessible habitat of medaka, it is possible to collect, analyze, and evaluate new wild catches and establish newly inbred strains.

From 1913 onwards, medaka was used to show Mendelian inheritance in vertebrates and in 1921 it was the first vertebrate in which crossing over between the X and Y chromosomes was detected (Toyama 1916; Aida 1921). In Japan there are two divergent wild populations of medaka separated by the Japanese Alps dividing the main island of Honshu (the Northern and Southern populations, Figure 1A) (Ishikawa *et al.* 1999; Takehana *et al.* 2003; Setiamarga *et al.* 2009; Asai *et al.* 2011). These two populations are not in sympatry (*i.e.*, do not overlap in the wild) and have many different phenotypic features; they can, however, produce fertile offspring when mated in the laboratory (Kimura *et al.* 2007). A critical feature of medaka laboratory husbandry has been the routine inbreeding of wild individuals from the Southern medaka population to isogenic strains pioneered by Hyodo-Taguchi in the 1980s (Hyodo-Taguchi 1980, 1990). Some of these strains are now in their 80th brother-sister mating, and importantly, there are routine protocols for creating an inbred strain from the wild. At least eight isogenic strains derived from single wild catches are available from the medaka NBRP stock center (Sasado *et al.* 2010). Furthermore, the availability of standard transgenesis protocols (Rembold *et al.* 2006), mutant lines (Furutani-Seiki *et al.* 2004), a 700-Mb reference genome sequence combined with a detailed linkage map (Kasahara *et al.* 2007), and tools for enhancer and chromatin analysis (Sasaki *et al.* 2009; Mongin *et al.* 2011) make medaka a powerful vertebrate organism for developmental and molecular studies (The Encode Project Consortium 2012).

**Figure 1:**
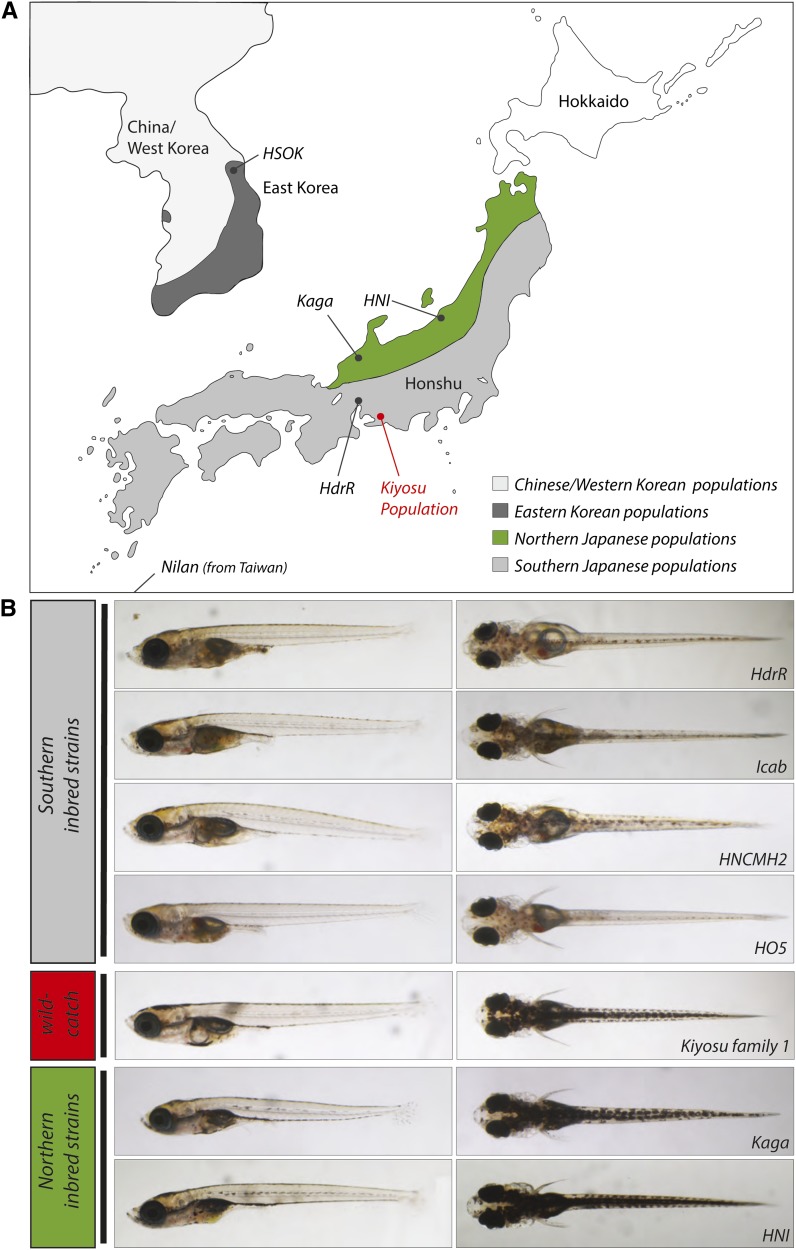
Geographical localization of the sampling sites for inbred strains and the Kiyosu population. (A) Medaka consists of four major populations in East Asia. The Nilan strain comes from Ilan-City, Taiwan, and is a representative of the Chinese/West Korean population; the HSOK strain is from Sokcho-City, Gangweon-do, Korea, and is a member of the East Korean population. The Japanese populations are divided by the Japanese Alps into Northern and Southern strains. Whereas Kaga and HNI belong to the Northern populations, HdrR is the Southern reference strain that was used for the Medaka genome sequencing project (Kasahara et al., 2007). In red is the site in Kiyosu where the founders for the inbreeding panel were sampled (see *Materials and Methods* for the GPS location). (B) Representative pictures of lateral and dorsal views of 10 days’ postfertilization-old larvae of the different Japanese inbred strains. Icab, HNCMH2, and HO5 are further Southern Japanese inbred strains whose original sampling site is not shown in (A).

Here, we characterize molecular, genetic, and phenotypic variation in a single Southern Japanese population of medaka to assess its suitability to found a near-isogenic wild panel. For comparison, we characterize a number of existing inbred strains, including the “reference” HdrR strain, and explore the population genomics of this species. In addition, we set the groundwork for routine high-throughput phenotyping of medaka strains to quantify morphometric features, which is one of many possible traits that can be measured in this vertebrate. On the basis of these results, we have begun the inbreeding cycles of 200 lines for establishment of a medaka inbred isogenic panel.

## Materials and Methods

### Fish stocks and husbandry

#### National Institute for Basic Biology (NIBB), Japan:

The Kiyosu population was sampled at a location with the following GPS coordinates: 34.780885° N, 137.347818° E (Kiyosu, Toyohashi, Aichi Prefecture). The Takashi Hongo population (showing mitotypes indicative of a human-mediated dispersal and not included in further analyses) was sampled 8.4 km (5.2 miles) to the southeast at 34.715135° N, 137.392623° E. The HdrR inbred line was generated from the original d-rR strain (Yamamoto 1953), which was established from commercially available orange-red and white varieties with unknown sampling sites. Phylogenetic analysis of the *cyt b* gene has suggested that HdrR belongs to the Southern Japanese population as has been confirmed here. The HNI inbred line is established from a wild population collected in Niigata city, Niigata prefecture, Japan. The Kaga inbred line is established from a wild population collected in Kaga, Ishikawa Prefecture, Japan. The HNI and Kaga lines belong to the Northern Japanese population. The HSOK inbred line is established from a wild strain of the West Korean population from Sokcho, Korea. The Nilan strain, which is not fully inbred (F16) is established from a wild population collected in Ilan, Taiwan, and belongs to the China-West Korean population. The precise GPS coordinates for each inbred line are not recorded. Classification of each inbred line to each population is according to Takehana *et al.* (2003, 2004). The numbers of brother-sister matings in the HdrR, HNI, Kaga, HSOK, and Nilan strains used for genome resequencing are F80, F70, F35, F30, and F16, respectively. The two additional inbred lines used in morphometric analyses, Icab and HNCMH2, were purchased from Carolina Biological Supply (Burlington, NC); these lines were derived from Southern Japanese populations through more than 20 generations of inbreeding. All inbred lines have been maintained by brother-sister mating. All procedures in these experiments were carried out by permission number 10A007 by the experimental animal committee at the NIBB.

#### Karslruhe Institute of Technology, Germany:

The following strains from the NIBB were kept as described previously under 14-hr light/10-hr dark conditions (Loosli *et al.* 2000): HdrR, Icab, HNCMH2, HO5, Kaga, HNI, and Kiyosu. Animal husbandry and experimental procedures were performed in accordance with the German law on Animal Protection and were approved by Local Animal-Protection Committee (Regierungspräsidium Karlsruhe, Az.35-9185.64).

### Genomic DNA extraction

Genomic DNA was extracted from one male specimen of each inbred strain at the age of about 8 weeks using Phenol/Chloroform/Isomylalcohol and RNA was removed using RNase A (Fermentas). For analysis of the wild catches DNA was extracted from caudal fins.

### Mitotyping

Polymerase chain reaction (PCR)-restriction fragment length polymorphism analysis of the mitochondrial cytochrome B gene was performed as described in Takehana *et al.* (2003). In brief, a 1241-bp segment including the complete cytochrome B gene was amplified, amplicons were digested with five restriction endonucleases (*Hae*III, *Mbo*I, *Msp*I, *Rsa*I, and *Taq*I), and mitotypes assigned by inspection.

### D-loop sequencing

D-loop sequencing was performed as described in Katsumura *et al.* (2009). The 616-bp region of the mitochondrial D-loop was amplified from caudal fin clip genomic DNA by PCR using the following pair of primers (5′ to 3′): Medaka_D-loop_F1: CCCAAAGCCAGGATTCTAA; Medaka_D-loop_R1: AACCCCCACGATTTTTGTC. Sequences were determined on both strands and then trimmed to 508 bp for comparison and aligned and analyzed with Geneious Pro 5.3.4 software (Biomatters).

### Design and analysis of microsatellite markers

To identify potential microsatellite regions, we compared the HdrR and the HNI genome by using Sputnik (Katsumura *et al.* 2009) and identified regions with a maximal repeat unit length of 2 and the minimum length of simple sequence repeat set to 20. We designed primers with primer3plus (Untergasser *et al.* 2007) to amplify a region of about 200 bp flanking the microsatellite repeat. We searched for one marker per chromosome but finally settled on nine high-quality microsatellite markers. Alleles were annotated from chromatograms after PCR amplification with fluorescently labeled oligonucleotides using GeneMarker software (Softgenetics). For a complete list of primers, see Table S1.

### Inbreeding coefficient calculation

The Inbreeding coefficient F was calculated (in a single population) as F = 1 − (*HOBS* / HEXP) = (*HEXP* – HOBS) / HEXP, where HOBS is the observed heterozygosity and HEXP is the expected heterozygosity calculated on the assumption of random mating.

### Short-read sequencing, reference assembly “patching,” and single-nucleotide polymorphism (SNP) calling

Eight paired-end libraries (insert size 300 bp) and three mate-pair libraries (insert size 3K) from the HdrR reference strain were sequenced using an Illumina HiSeq machine to a median coverage of 144X. Reads were aligned using Bowtie2 (Langmead and Salzberg 2012) and SNPs called using SAMtools (Li *et al.* 2009). Raw sequence reads and annotations were submitted to DDBJ (accession DRA000588). The HdrR reference genome sequence was “patched” using base differences passing a quality threshold of 100 and with additional bases not defined in the reference (*i.e.*, originally listed as ‘N’). However, when using this patched sequence as reference for calling SNPs in other inbred lines, we noticed that a small number of SNP calls (~0.14–0.2% total) were consistent with the base identified from the short-read HdrR sequence at quality scores below this threshold. The use of an unthresholded HdrR dataset as the reference resolved this problem, albeit resulting in a marginal addition of 0.01–0.017% SNPs that were consistent with the original reference. On the balance of the false-positive and false-negative rates estimated this way, we opted for using the unthresholded “patched” reference for calling SNPs in all other strains. Furthermore, we supplemented the final thresholded patched sequence with 89,227 bases that were consistent with the other four inbred line sequences and submitted the final sequence as a Third-Party Annotation record to the European Nucleotide Archive (ENA; HF933207-HF933230). For 41 loci, SNPs were validated by direct sequencing after PCR amplification using the primers listed in Table S5. Samples from the other inbred strains were sequenced with a combination of paired-end and mate-pair Illumina sequencing to a median coverage of 108-125X. Reads were aligned using Bowtie2 and SNPs called using SAMtools based on the patched assembly. Refer to Table S6 for details.

A total of 24 wild individuals of the Southern population in eight mother-father-offspring trios were sequenced using paired-end sequencing to a median coverage of 9X. The sequence was aligned to the patched reference sequence and SNPs were called using SAMtools. Alleles segregating within the population were then phased and annotated taking into account family structure using TrioCaller (Chen *et al.* 2013). SNPs were filtered by the empirical *r^2^* between inferred and expected values (*r^2^* ≥ 0.6) and the observed Mendelian error (ERATE ≤ 0.1). Raw reads were submitted to ENA (accession no. ERP001016).

### Analysis of protein-coding mutations

Exon mappings for all regions mapping to chromosome scaffolds in the medaka 2005 reference assembly were obtained from Ensembl. SNPs were annotated as leading to either nonsense, stop-codon, missense, or synonymous substitutions (note that no stop codon mutations were in fact observed). Ambiguities in this annotation due to overlapping transcripts were resolved in this order of preference (*i.e.*, if a SNP produced a nonsense mutation in at least one overlapping transcript, it was annotated as such, and so on). Expected relative frequencies of each coding mutation type were estimated on the basis of all theoretically possible single-nucleotide substitutions at each codon and the observed frequencies of each codon in the medaka exome.

### Phylogenetic analysis

Phylogenetic relationships between the wild Southern population, five inbred strains, and stickleback were assessed using a neighbor-joining algorithm based on Kimura two-parameter distances implemented in PHYLYP, *i.e.*, the *PHYL*ogeny *I*nference *P*ackage (Retief 2000). Genotypes for the wild population were called using a “majority vote” across the 48 haplotypes. Differences between medaka samples and stickleback were too large to estimate distances. Hence, the length of the stickleback branch shown in Figure 3 is an underestimate. The tree was rooted using the midpoint method and plotted using the T-REX online tool (Boc *et al.* 2012). The relationships between the wild samples and the reference strain (Figure 3B) were analyzed in R using a hierarchical clustering with Gower distances (Struyf *et al.* 1997) based on the SAMtools genotypes.

### Estimation of linkage disequilibrium (LD)

LD (*r^2^*) was computed from the TrioCaller SNPs from the 16 wild Southern trio founders as well as in 16 parents from human trios sequenced and annotated by Complete Genomics (Drmanac *et al.* 2010). Computations were performed using VCFtools (Danecek *et al.* 2011) with the following options: –ld-window-bp 50000, –max-alleles 2, –min-alleles 2, –min-r2 0.001, –geno 0.8. Haplotype blocks in the 16 medaka founders were called using HaploView (Barrett *et al.* 2005) using the default parameters for the method described by Gabriel *et al.* (2002). Trials involving varying the minor allele frequency cut-off and the fraction of informative markers required to be in strong LD indicated that block size was relatively stable to these parameters (data not shown).

### Population size history analysis

To estimate changes in population size over time, we used the method of Li and Durbin (2011), implemented in the software package psmc. The following options were used with the psmc executable: -N25 -t15 -r5 -p “4+25*2+4+6,” ensuring that the predicted number of recombinations occurring in each specified time interval were sufficiently high (>10) to prevent overfitting. The output was scaled assuming a doubling time of 0.67 years per generation and a mutation rate of 2.5 × 10^−8^. Results for all individuals were combined by binning the results into log_10_ time intervals of length 0.1.

### Detection of signals of recent positive selection

A method proposed by Voight *et al.* (2006) and implemented in the R package rehh (Gautier and Vitalis 2012) was used. The standardized output statistic (integrated haplotype score, iHS) was centered around zero, as expected, and was filtered using the following criteria to identify robust and consistent signals: (1) |iHS| ≥ 0.1 (corresponding to the top 0.05% of the distribution); (2) within a 20-kb window surrounding each SNP: (a) standard deviation (iHS) ≤ 0.34 across all SNPs (bottom 50% of the distribution), (b) the number of SNPs with an iHS of at least 80% max(|iHS|) is at least 4, and (c) the number of SNPs with an iHS of at least 80% max(|iHS|) constitutes at least 10% of all SNPs in the window. Thus, clusters of SNPs with highly negative iHS values (indicative of derived alleles under recent positive selection) are detected. None of the positive iHS values (potentially corresponding to hitchhiker alleles or a “switched” directionality of selection (Voight *et al.* 2006) passed this filter.

### Phenotyping of morphometric features

Morphometric features of four Southern (HdrR, Icab, HNCMH2, HO5), two Northern (Kaga, HNI) medaka lines, and the F1 offspring of two Kiyosu brother-sister mating pairs (*i.e.*, one generation of brother-sister inbreeding) were analyzed. All embryos were raised under identical environmental conditions. The embryos were imaged at 10 days post fertilization by mounting them in 85% glycerol. Imaging was performed using a Leica MZ 16 FA stereomicroscope with a Planapo 1.0× objective and 20× zoom factor. To extract morphological features, a manual and an automated algorithm were developed. Although the manual algorithm allows the user to select features of interest, the automated algorithm automatically segments predefined landmarks. It is based on contour detection, morphological image filtering; and connected component labeling. The automated algorithm was implemented in MATLAB, and its accuracy was verified with the manual algorithm.

### Assessment of heritability

Broad-sense heritability was calculated as the proportion of variance assigned to strain factors in an analysis of variance. The different morphometric measurements were divided by body length to remove the strongest source of variance. The ratios of the other measurements were analyzed using the *aov* function in R, and the proportion of variance explained determined as the square of the residuals of the strain factor over the total sum of squares. Between 50 and 100 individuals were analyzed in each strain.

### Data submissions

Whole-genome sequencing of the 5 medaka inbred strains was submitted to the DNA Data Bank of Japan under accession DRA000588. Whole-genome sequencing of the eight medaka wild catch trios from the Kiyosu population was submitted to the ENA under accession ERP001016. The revised genome sequence was submitted to ENA as accessions HF933207-HF933230. The SNPs discovered are currently being submitted to the Single Nucleotide Polymorphism Database (dbSNP), and identifiers will be available.

## Results

### Refining the medaka reference genome sequence and genotyping the existing inbred lines

In preparation for the analysis of a wild population, we used high-throughput sequencing to assess the genetic diversity across the existing inbred strains, including the HdrR strain that was used to generate the medaka reference sequence.

### Refining the medaka reference genome sequence by high-throughput sequencing

The current medaka reference assembly (Kasahara *et al.* 2007) is based on Sanger sequencing of the HdrR inbred strain derived from a Southern Japanese population. To verify and refine the reference genome sequence, we applied paired-end high throughput short read sequencing to genomic DNA from the HdrR inbred line, to a median depth of 144× (see *Materials and Methods* for details). Consistent with the high quality expected from the reference sequence, we identified 184,318 single nucleotides (0.026% of bases) unresolved in the reference assembly (labeled as ‘N’) and 102,432 single nucleotides (0.014% of bases) that were discrepant between the reference genome assembly and the newly obtained sequence above our quality threshold. Further investigation showed that revision of discrepant bases in the reference sequence toward the newly determined sequence resulted in 89% of revised sites matching all individuals in an independently sequenced wild Southern Japanese population (described in *Selecting and genotypically characterizing a founder population from Southern Japan*), with 96% matching at least one of the analyzed individuals in this population. In addition, revision of these discrepant positions resulted in a striking 2.5-fold increase in consistency with the presumed ancestral allele as determined by comparison to stickleback. Using direct sequencing after PCR amplification, we were able to confirm the new sequence for 67.5% (27) of a set of 40 discrepant bases, whereas the additional 32.5% (13/40) showed traces of both possible sequences and could not be disambiguated. These observations support the hypothesis that these divergent positions represent errors in the original reference sequence. The discrepancies span 490 genes and suggest 804 alternative amino acid sequences and 11 previously unmapped stop codons.

Given the large depth of coverage of the newly obtained genome sequence, and the successful verification of a subset of bases in conflict, we accounted for revisions to the HdrR reference sequence based on the short read data in the subsequent analysis of all other samples in this study. Further refining the reference calls based on consistency with other inbred strains (sequenced as described in *Whole-genome genotypic characterization of existing medaka inbred lines*), we produced a revised reference assembly, which we submitted to the ENA as a Third-Party Annotation (accession nos. HF933207-HF933230; see *Materials and Methods* for more details).

### Whole-genome genotypic characterization of existing medaka inbred lines

**Figure 2:**
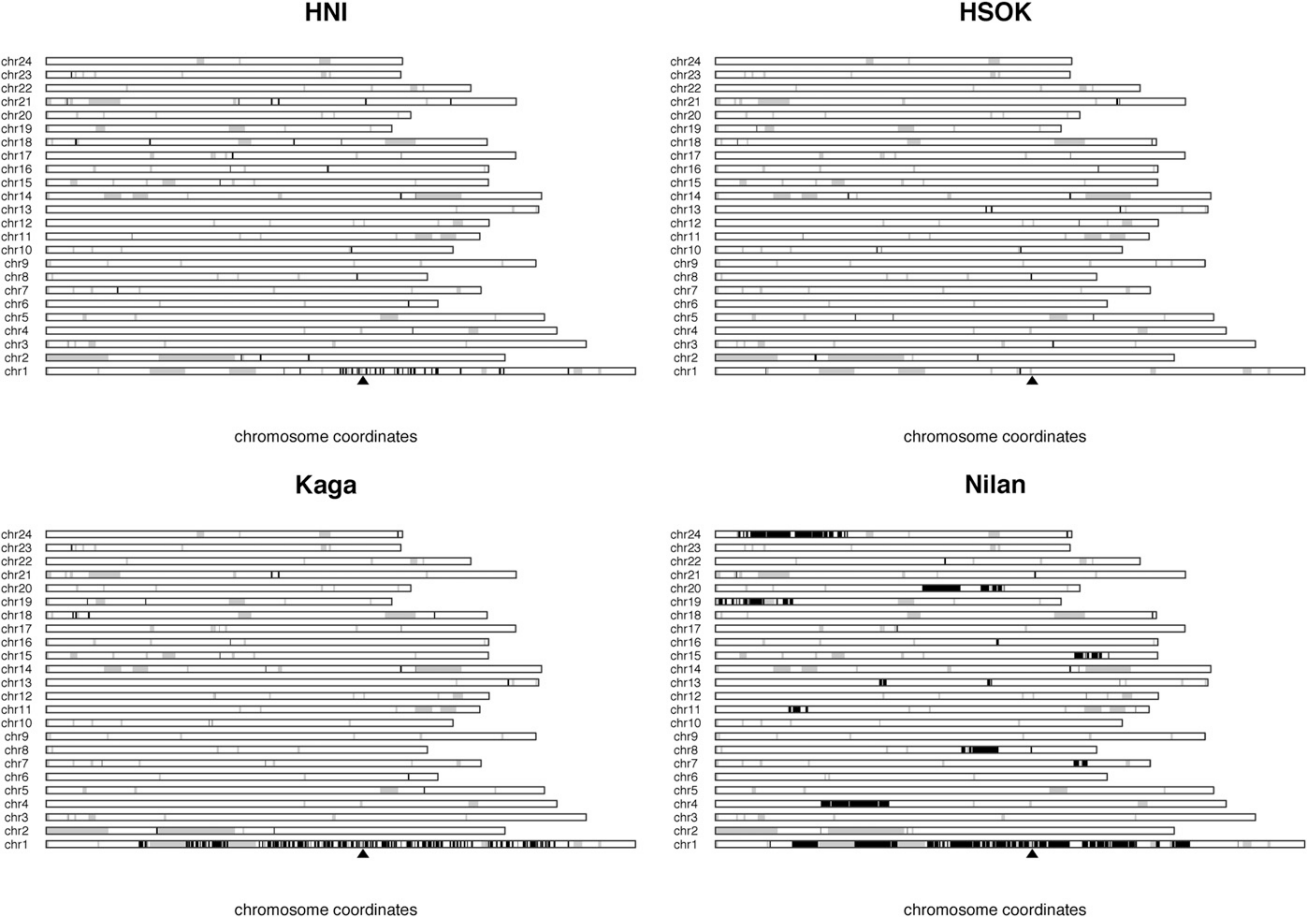
Levels of heterozygosity in medaka inbred lines. Four plots showing regions of heterozygosity consistent with a wild origin in the HNI, HSOK, Kaga, and Nilan inbred strains. Black blocks show windows of heterozygosity >7.1 × 10^−4^. At this threshold, 90% of the genome in the wild catches is classified as heterozygous. Gray blocks are assembly gaps. Chromosome 1 in medaka is the known location of the sex determination locus as indicated by the black arrow heads (Katsumura *et al.* 2009).

We next used high-throughput short read sequencing to characterize the genotypes of four additional inbred lines derived from three different locations: Northern Japan [HNI and Kaga (Hyodo-Taguchi 1980; Hyodo-Taguchi 1990; Loosli *et al.* 2000)], South Korea [HSOK (Sasado *et al.* 2010)], and Taiwan [Nilan (Sasado *et al.* 2010)]. We discovered 20,522,880 single-nucleotide variations within the four strains with divergence from the reference strain at 1.3–1.37% depending on the strain (Table 1), confirming the highly polymorphic nature of medaka consistent with the broad geographical spread of its natural habitat (Setiamarga *et al.* 2009). The inbred lines are predominantly highly homozygous (Figure 2; 0.1% of divergent sites are heterozygous) with most inbred strains having heterozygosity levels around 100-fold lower (average around 3 × 10^−5^) than a wild catch (1.5 × 10^−3^) (Table 1) and, with the exception of the Nilan strain and chromosome 1, there are only a few blocks of high heterozygosity consistent with a wild heterozygous origin. The Nilan strain is not as inbred as the others (heterozygosity at 2 × 10^−4^, more blocks of wild-like heterozygosity, 1% of divergent sites are heterozygous), as expected from far fewer cycles of inbreeding (Nilan, 16 inbreeding generations; Kaga, 35; HSOK, 30; HNI, 70). Chromosome 1 harbors the sex determination locus, and as the individuals sequenced for Nilan, Kaga, and HNI were male, the stretch of heterozygosity is centered on the known sex determination locus (Figure 2). We conclude that inbreeding in medaka can create highly homozygous individuals, with the expected exception of the sex chromosome.

**Table 1:** Sequence variation in four medaka inbred strains and the Kiyosu wild population from Southern Japan compared with the reference HdrR strain

Strain	SNPs	(as %)	Genes With at Least One Variant	Number of Missense SNPs	Number of Nonsense SNPs
Nilan	9,078,050	1.3%	16,386	86,545	679
HSOK	9,583,441	1.37%	16,590	83,261	755
Kaga	9,590,114	1.37%	16,603	75,187	742
HNI	9,522,479	1.36%	16,612	74,921	711
Total inbreds	20,522,880	2.93%	16,925	175,856	1,682
Kiyosu population	4,797,962	0.69%	15,727	46,639	611

Numbers are given for pairwise comparison with HdrR (the reference) and compared with the 17,442 genes on chromosome scaffolds of the 19,686 genes in the medaka reference gene annotation. The counts include both homozygous and heterozygous sites in each inbred strain (0.1% of divergent sites are heterozygous for Hsok, Kaga, and HNI; 1% for Nilan). The results for the Kiyosu population include SNPs detected in at least a single individual in either homozygote or heterozygote. See *Materials and Methods* for details on the SNP quality thresholds used, the sequencing coverage and the genotyping methods used for the inbred strains and the Kiyosu population. SNP, single-nucleotide polymorphism.

**Figure 3:**
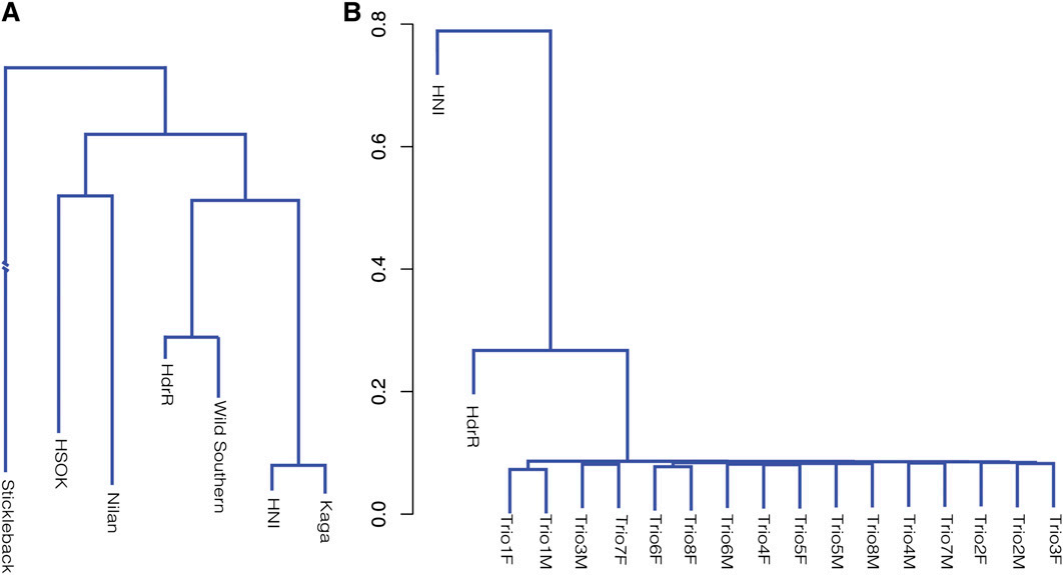
Phylogenetic relationships of medaka inbred strains and the Kiyosu population. (A) Phylogenetic relationships between the wild Southern population, five inbred strains, and stickleback based on high-throughput genome sequencing. PHYLIP clustering of inbred strains using a neighbor-joining algorithm based on Kimura two-parameter distances is shown. Wild Southern is a synthetic sample based on the sequencing of the Kiyosu population and determining bases using majority vote across 48 haplotypes. Differences between medaka samples and stickleback were too large to estimate distances. (B) Hierarchical clustering with Gower distances of genotypes of Kiyosu population founder samples, and representative Southern and Northern strains.

Of the 20,522,880 SNPs detected across the inbred lines, 529,009 were mapped to 16,925 of 17,442 medaka genes on chromosome contigs, of which 33.5% led to changes in amino acid sequence, including 175,856 missense mutations in 15,821 genes and 1682 nonsense mutations (Table 1). As expected, missense and particularly nonsense mutations were strongly depleted in favor of synonymous SNPs compared with random expectation, presumably due to purifying selection: 33.2% (175,856) missense mutations *vs.* 70.8% expected at random and ~0.3% (1682) nonsense mutations *vs.* ~4.4% expected at random (binomial test *P* < 1 × 10^−300^ in both cases; expected values were estimated accounting for codon frequencies in the medaka exome). Of these nonsense mutations, we confirmed 15 of 16 selected loci within the Kaga, HNI, and Nilan strains by targeted PCR and sequencing. In addition, we validated 9 of 10 noncoding Kaga strain SNPs, confirming the high reliability of our sequence data. The phylogenetic relationship between the strains based on the sequencing data (Figure 3A) was consistent with the geographical localization of the sampling sites of the inbred strains (Figure 1A) and earlier data based on a limited number of SNPs (Kasahara *et al.* 2007).

### Selecting and genotypically characterizing a founder population from Southern Japan

#### Selecting the founder population:

We set out to identify a genetically diverse, stable medaka founder population for the establishment of a panel of further isogenic lines. For this purpose we sampled irrigation canals in the Takashi Hongo and the Kiyosu areas near Toyohashi (Aichi Prefecture) in July 2010. We first analyzed the mitotype of 50 and 109 individuals from the two sites, respectively, as described by Takehana *et al.* (2003). The most likely source of population structure in these samples is human-mediated dispersal of fish, *e.g.*, fish discarded from aquariums. We detected an unusual mitotype in the Takashi Hongo population, normally present only in Northern Japanese medaka and that is a likely marker of human-mediated dispersal. Hence, we decided to focus on the Kiyosu population in further analysis.

#### Estimating the population diversity by sequencing variable genomic regions:

We sequenced the highly variable D-loop region of the mitochondrial cytochrome B gene in 105 Kiyosu individuals and detected eight different sequence variants. This degree of D-loop sequence diversity was comparable with an earlier study in which Katsumura *et al.* (2009) detected 11 sequence types in 124 individuals in the most diverse and three types in 159 individuals in the least diverse of three wild medaka populations. Compared with the established medaka inbred strains, the Kiyosu population clustered with the southern HdrR, HO5, and HNCMH2 strains as expected (Figure S1). We analyzed microsatellite markers designed for 9 of the 24 medaka linkage groups (Table S1) and detected up to 21 different alleles within 105 individuals (Table S2). An ideal founder population for establishing a medaka population genomics panel would be without significant population structure. We calculated inbreeding coefficients (F) from these microsatellite allele frequencies. Negative values of F indicate excess heterozygosity (outbreeding), whereas positive values indicate heterozygote deficiency (inbreeding) compared with Hardy-Weinberg expectations. In the case of our founder population, F ranged from −0.188 to 0.203 with seven positive and two negative values and an average of 0.107. Based on this preliminary analysis, we concluded that there is a tolerable degree of inbreeding within our population and decided to proceed with deeper analysis.

#### Genome-wide sequencing of eight trios from the founder population:

To analyze the Kiyosu population in greater detail, eight mating pairs and their first-generation progeny (*i.e.*, eight trios) were selected. The genomes of these individuals were profiled by the use of high-throughput short read sequencing, with ~9X mean depth of coverage per line and compared with the Southern Japanese reference HdrR sequence. Approximately 23% of variants detected were monoallelic across Kiyosu, distinguishing this population from the reference HdrR strain. Using a trio-aware algorithm [TrioCaller (Chen *et al.* 2013)], we scored and phased the remaining 77% of variants, giving a total of 4,797,962 SNPs segregating within the Kiyosu population (Table 1). Of these, 107,950 mapped to 15,727 coding regions, with 59,700 synonymous, 47,639 missense, and 611 nonsense substitutions. As in the inbred lines, the latter two mutation types were considerably depleted in favor of synonymous SNPs compared with random expectation, as expected under purifying selection (44.1% missense mutations *vs.* 70.8% expected at random and 0.56% nonsense mutations *vs.* 4.4% expected at random, respectively, binomial test *P* < 1 × 10^−300^ in both cases). Similar observations have been made recently in other population surveys of species such as Arabidopsis and human (Gan *et al.* 2011; MacArthur *et al.* 2012). We note that the proportion of nonsense mutations observed in the Kiyosu population is nearly twice as high as in the inbred lines (0.56% in Kiyosu compared with ~0.3% in the inbred strains). One explanation for this is the greater tolerability of nonsense mutations when present in a heterozygote. Indeed, only 228 of the 611 (37.3%) nonsense mutations detected in the Kiyosu wild population are homozygous in at least one individual.

This polymorphism set provides the opportunity for a far more powerful test of population structure than the aforementioned microsatellite approach. Clustering the sample genotype differences by distance metric, we found that the parental genotypes were largely equidistant from each other in the tree without visible structure (Figure 3B). As expected, the wild individuals clustered closest to each other near the Southern reference strain HdrR collected in the same region of Japan, confirming the results from aforementioned directed genotyping.

### Linkage disequilibrium estimates suggest medaka as an attractive model for genome-wide association studies

**Figure 4:**
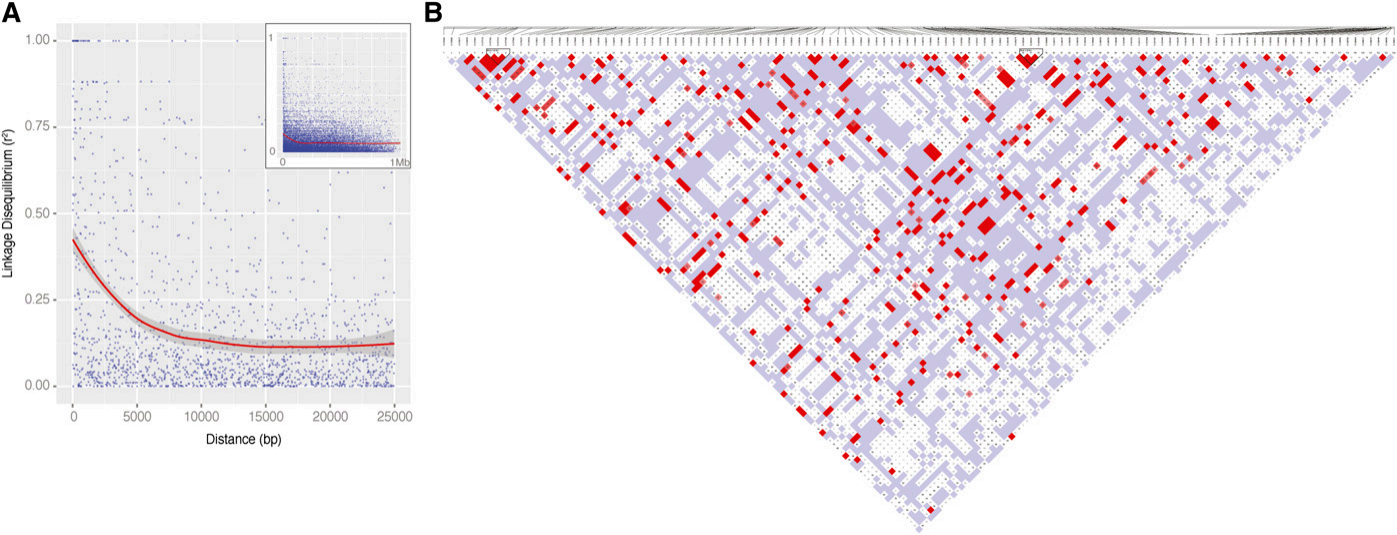
Linkage disequilibrium (LD) in the Kiyosu medaka population. (A) Decay of LD for a sample of medaka SNPs. LD (*r^2^*) was calculated for all SNP pairs in 1-Mb windows overlapping by 500 kb across medaka chromosome 1, and r2 was plotted against distance for a sample of 0.03% (21976) of pairs. The main plot shows a blow-up of distances between 0 and 25 kb, with data for pairs of SNPs up to 1 Mb apart shown in the inset region. Multiple sampling and analysis of other chromosomes gave similar results (not shown). (B) Pairwise LD plot for a representative region of chromosome 11. Pairwise LD (D′) for the 25-kb region 11:375000−399999 as displayed in Haploview is shown. The plot shows the LD of pairs of SNPs by squares joining the two SNPs via the diagonal and is color coded as described for the standard LD color scheme at http://www.broadinstitute.org/science/programs/medical-and-population-genetics/haploview/ld-display such that pairs of SNPs in LD at D′ = 1 are colored blue at LOD <2 and red at LOD ≥ 2, whereas those with D′ < 1 are white at LOD < 2 and shades of red/pink at LOD ≥ 2.

Linkage disequilibrium (LD) is the deviation from independent segregation of alleles between loci and is dependent on recombination rate and population history. The extent of LD is an important parameter for mapping genotype-phenotype associations within a population. We estimated the LD for the medaka Kiyosu population from the 16 parental sample genotypes expressed as *r^2^*, which is robust to smaller sample sizes and expresses the key relationship in association mapping. Figure 4A illustrates that median *r^2^* between pairs of loci gradually drops with increasing distance, reaching a minimum at a distance of approximately 12.5 kb. To compare this with human LD estimates obtained in the same setting, we selected 16 independent parents from trios sequenced by Complete Genomics (Drmanac *et al.* 2010). In this human population *r^2^* stabilizes at ~37 kb, which is ~3 times larger. For our medaka data, ~85% of SNP pairs with *r^2^* > 0.8 mapped to the same gene, with ~37% mapping to the same exon, indicating that fine mapping in medaka should largely be possible to the resolution of single genes and frequently even to a single exon. For comparison, only 56% and 29% of the equivalent SNP pairs mapped to the same gene and exon in the equivalent human population, respectively. We also estimated haplotype block size across 15 of the medaka chromosomes by using the method of Gabriel *et al.* (2002). The mean haplotype block size was 712 bp (median 259 bp) with a maximum of ~78 kb. Figure 4B shows the LD map of a representative region of chromosome 11. We conclude from the detailed genetic characterization of this Kiyosu population sample that these fish will be suitable for establishment of a population genomics resource.

### Whole-genome polymorphism data provide insights into medaka population history and evolution

The determination of polymorphism data in a wild medaka population allows us to examine a number of questions in medaka population genetics, such as probe for interbreeding between different medaka populations, infer population history, and identify loci that are likely to undergo a recent positive selection.

#### Probing for potential interbreeding between medaka populations:

Phylogenetically, the Northern Japanese medaka strains lie between the Southern Japanese and the Chinese and Korean medaka populations, which form an outgroup to the two Japanese clades (Takehana *et al.* 2004). There are several phenotypes unique to the Northern strains compared with all other medaka, including a requirement for shallow water tanks for good viability, and differences in behavior, pigmentation (Figure 1B) as well as in morphometric parameters (as will be presented in *Morphometric analysis as a promising tool to assess medaka phenotypic diversity*). Recently these divergent phenotypes have even led to suggestion that the Northern and Southern medaka populations are distinct species (Asai *et al.* 2011).

To clarify the genetic relationship between the Northern and Southern Japanese medaka clades, we investigated potential wild interbreeding events drawing information from the Kiyosu population and the inbred strains. When interbreeding occurs, genotypes will be exchanged between populations that have separated some time ago, events that are referred to as introgression. Recent studies show that whole-genome data can illuminate ancient introgression events that are not visible by methods typing more limited numbers of loci (Green *et al.* 2010; Ishiguro *et al.* 2012; Meyer *et al.* 2012).

The test for introgression proposed by Durand *et al.* (2011) is based on the expectation that the ratio of ancestral alleles (*e.g.*, those that medaka has in common with stickleback) to derived alleles is likely to be equal across populations descending from a common ancestor (*e.g.*, two Southern populations). If, however, one of these populations exchanged genes (introgressed) with a third population that is further away on the phylogenetic tree (*e.g.*, a Northern population), this symmetry would be perturbed. Specifically, at positions where this Northern population has derived alleles, the Southern population that introgressed with it is more likely to also have derived alleles compared with its nonintrogressed Southern counterpart.

Using this approach, we found limited evidence of introgression between the Northern (HNI) and Southern Japanese samples. Surprisingly, however, the observed effect was stronger in the Southern inbred HdrR strain than in the Kiyosu population. Since these two samples share a high sequence similarity, we asked whether the result for the Kiyosu population is in fact a “ghost population” artifact (Durand *et al.* 2011) of its similarity with HdrR. Indeed, when the analysis was limited to just SNPs that differentiate the Kiyosu population from HdrR, no evidence for the introgression of Kiyosu with HNI was obtained (Table S3).

Applying a similar logic, we found evidence of introgression between the Taiwanese (Nilan) inbred strain and HdrR, but less so between Nilan and the Kiyosu population (Table S3).

The results based on the Kiyosu population and the Northern strains indicate that there was either extremely limited or no interbreeding between the Northern and Southern medaka lineages (at least to the point of the most recent common ancestor of the wild Kiyosu population and the Northern strains). At the same time, we have obtained evidence for introgression between pairs of some laboratory lines. Although these observations could reflect the population history of the wild populations from which the inbred lines were sampled, they may also be the results of historical laboratory interbreeding. Further, well sampled, wild individual sequences from Northern Japan, Korea, and Taiwan would be needed to fully resolve this issue.

#### The population history of Southern Japanese medaka:

**Figure 5:**
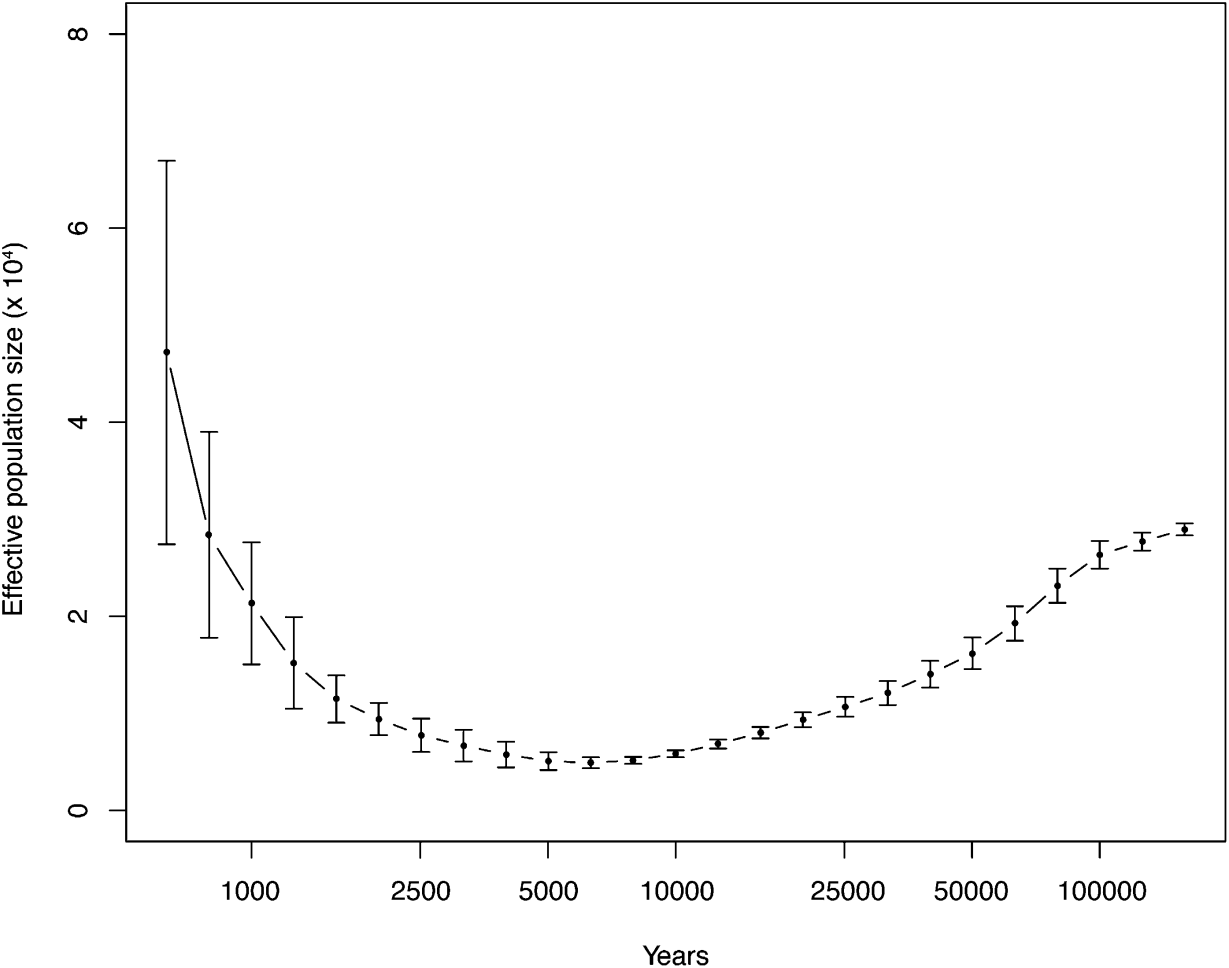
Distribution of population size estimates from the Kiyosu individuals. Population size estimates over time were calculated using the psmc package for the founder individuals in the Kiyosu wild catch as described in *Materials and Methods*. The graph shows the distribution of estimates after combining by binning.

It has been shown in humans, pigs, and giant pandas that it is possible to determine a population history in terms of effective population size from a single diploid individual based on the local density of heterozygous sites (Li and Durbin 2011; Groenen *et al.* 2012; Zhao *et al.* 2013). This approach exploits the presence of many thousands of independent segments in each individual genome separated by ancestral recombination events. A Hidden Markov model integrates the uncertainty of segments to provide a consistent view of the effective population size as a function of time. We performed a similar analysis on all the individuals in our Kiyosu wild catch sample. Figure 5 shows the distribution of population size estimates from the Kiyosu individuals. Except for the expected variability of estimates for very recent history, the population history profile was consistent between the individuals and is a different shape from the other species analyzed previously. This profile suggests a very large older population that went through a relatively recent bottleneck approximately 10,000 years ago (assuming a position-wise mutation rate of 2.5 × 10^−8^ per generation, generation time = 1 year) followed by a modest expansion in recent history. In addition to its immediate significance for medaka evolution, this result highlights the applicability and reproducibility of effective population size over time estimation for single-individual diploid genomes of model organisms determined using short-read sequencing.

#### Evidence of recent selection in the Southern Japanese medaka population:

We have used the wild genotypes to look for patterns of recent positive selection in the Southern population. An established footprint of recent selection is unusually long haplotypes of low diversity surrounding the allele under selection, whereas the alternative allele(s) are associated with haplotypes of a more typical length (Sabeti *et al.* 2002). A metric termed extended haplotype homozygosity (EHH) describes this effect for a given locus representing the probability that two randomly chosen chromosomes carrying the core haplotype of interest are identical by descent for the entire interval from the core region to any point *x*. EHH thus detects the transmission of an extended haplotype without recombination (Sabeti *et al.* 2002). We applied an established statistical approach based on the ratio of EHH decay with distance (known as iHS for integrated haplotype score; Voight *et al*. 2006) for the ancestral and derived allele using the parental genotypes of the newly established Southern strain trios. After applying stringent iHS filters (see *Materials and Methods*), we found that 192 SNPs, most of which map to 12 broad domains, showed evidence for recent positive selection of the derived haplotype (Table 2). As expected, there were no cases with an unusually long ancestral haplotype at this cutoff. These domains contained 17 genes, and in three cases the implicated SNPs mapped to their coding regions [*thioredoxin reductase 3* (defense against oxidative stress), *kelch-like 10*, *solute carrier family 41*], causing nonsynonymous amino acid changes. Interestingly, nearly 20% of SNPs indicative of recent positive selection localized to highly conserved, potentially regulatory genomic regions (1.4-fold enrichment over random expectation, *P* = 0.03, Table S4). This indicates that recent selection events have resulted in potentially significant changes in the coding and noncoding sequence, paving the way for a further examination of their impact on the molecular function and physiology of the organism as whole.

**Table 2:** Regions under positive selection in the medaka genome

Chromosomal Region	Gene Symbol	Gene Description	Ensembl Gene ID	Location of indicative SNP(s)
5:22601619-22654959	*slc41a3*	Solute carrier family 41	ENSORLG00000011740	ORF (341: Met → Val, 354 Leu → Phe), 4 intronic
5:22601619-22654959	*chst13*	Carbohydrate (chondroitin 4) sulfotransferase 13	ENSORLG00000011752	3 intronic, ORF (syn)
5:23874411-24206161	*klhl10*	Kelch-like 10	ENSORLG00000011990	ORF (100: Lys → Asn; 353: Ala → Thr), 1 intronic
5:23874411-24206161	*txnrd3*	Thioredoxin reductase 3	ENSORLG00000011831	ORF (457: Val → Leu; syn), 4 intronic
5:23874411-24206161	*bin2*	Bridging integrator 2	ENSORLG00000011869	4 intronic
5:23874411-24206161	*wnt4*	Wingless-type MMTV integration site family member 4a	ENSORLG00000012025	2 intronic, 1 downstream
4:5331132-5332602	*lrrc8c*	Leucine rich repeat containing 8 family, member C	ENSORLG00000002275	3 intronic
7:22770082-22774016	*racgap1*	Rac GTPase activating protein 1	ENSORLG00000015540	1 upstream
14:2110873-2114262	*h2afy*	H2A histone family, member Y	ENSORLG00000000850	7 intronic
15:24520836-24521875	*col19a1*	Collagen, type XIX, alpha 1	ENSORLG00000010350	2 intronic
15:7870807-8785145	*ube2z*	Ubiquitin-conjugating enzyme E2Z	ENSORLG00000001771	5 intronic, ORF (syn)
15:7870807-8785145	*rims1*	Regulating synaptic membrane exocytosis 1	ENSORLG00000001748	5 intronic
18:484757-484758	Novel	Novel gene	ENSORLG00000002268	1 intronic
21:2870759-2888920	*slc39a10*	Solute carrier family 39 (zinc transporter), member 10	ENSORLG00000008760	5 intronic
1:9439527-9460390	NA	NA	NA	Gene desert
9:14217964-14319610	NA	NA	NA	Gene desert
11:25012896-25023149	NA	NA	NA	Gene desert

Chromosomal regions detected as under positive selection are given along with details of the gene(s) contained within the regions and the locations of the indicative SNP(s). SNP, single-nucleotide polymorphism; NA, not applicable.

### Morphometric analysis as a promising tool to assess medaka phenotypic diversity

**Figure 6:**
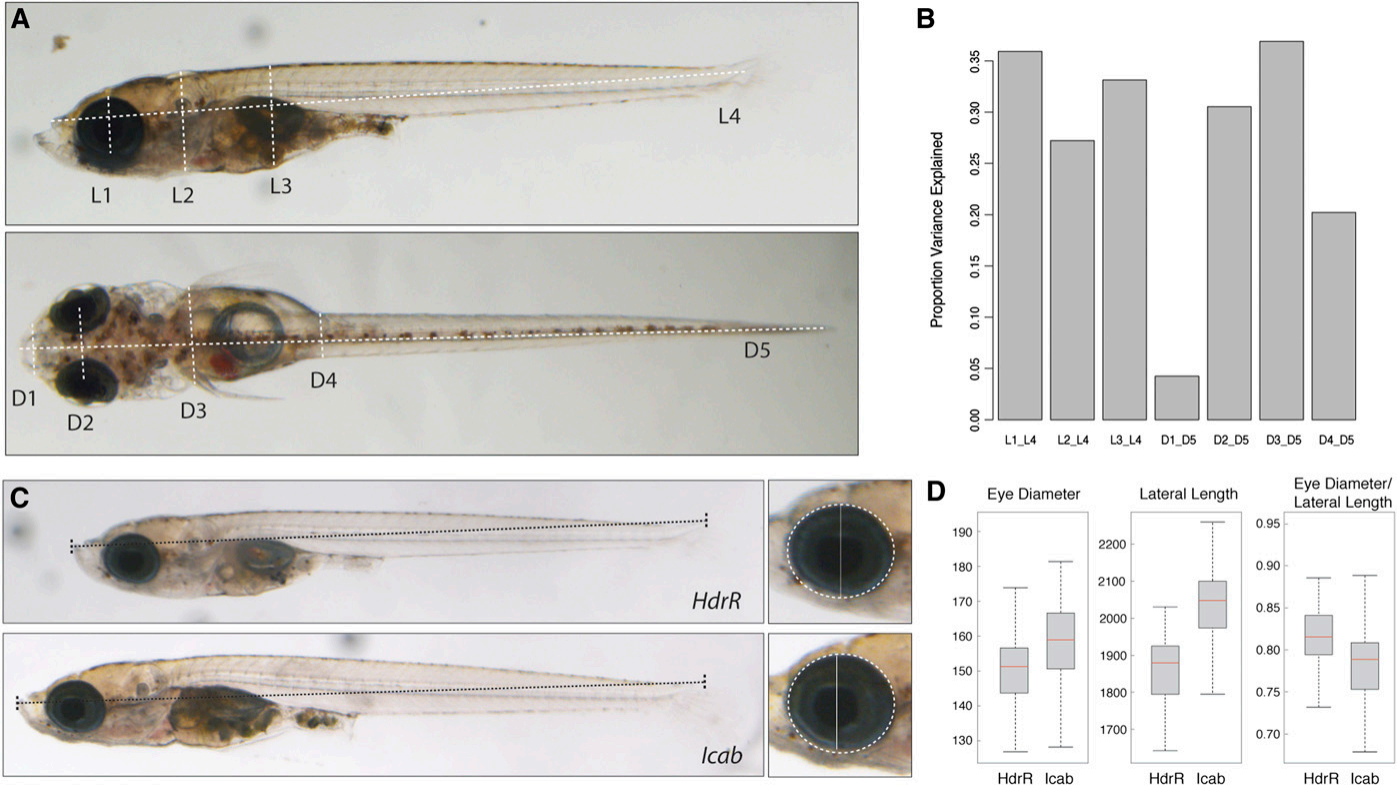
Morphometric analysis of medaka inbred strains. (A) Four lateral (L1-L4) and five dorsal (D1-D5) morphometric distances were extracted and analyzed for each inbred strain. (B) A bar chart showing the proportion of variance explained by the difference between strains as a fraction of the total variance. The variables are the measurements corrected by the appropriate body length measurement (L4 and D5, respectively). (C) Example pictures showing substantial differences in lateral length and eye diameter between fish from the two Southern inbred strains HdrR and Icab. (D) Box plots showing the distribution of eye diameter, lateral length, and the ratio between eye diameter and lateral length for HdrR and Icab. Eye diameter and lateral length: y-axis = value in pixel. HdrR (n = 70), Icab (n = 78).

An important feature of a population panel is that it is expected to exhibit appreciable phenotypic variation across individual lines. To characterize one set of phenotypes, we took advantage of the Southern inbred lines already in existence because these are likely to be representative of lines generated from the Kiyosu population, allowing us to easily characterize broad-sense heritability of phenotypes. We used light microscope-based imaging combined with an automatic annotation algorithm to derive a number of morphometric features across four Southern (HdrR, Icab, HNCMH2, HO5), two Northern strains (Kaga, HNI), and the Kiyosu population (Figure 6A, Figure S2, and Figure S3) from both lateral and dorsal viewpoints. The morphometric analysis of the six inbred lines shows reassuringly that the body length measurement was almost perfectly correlated between the two viewpoints. As expected in a fish, the majority of the variance between individuals correlates with body length, and so we normalized the remaining morphometric phenotypes by body length. Of the seven phenotypes analyzed, four show greater than 30% broad-sense heritability *i.e.*, the differences between strains explained 30% or more of the variance (Figure 6B). An example is shown in Figure 6C, where a fish from the HdrR strain has substantially smaller eyes relative to body length than the Icab strain (compare box plot in Figure 6D). The broad-sense heritability estimate is similar to analogous morphometric measurements between inbred mouse strains, suggesting that medaka populations have similar levels of phenotypic variation as observed between laboratory mouse strains. Many other established phenotypes that differ between medaka strains have been observed during the previous century of research on this fish (Hyodo-Taguchi 1990; Ishikawa *et al.* 1999; Kimura *et al.* 2007), and further work will be needed to examine the suitability of this population for each phenotype. Given the high diversity of alleles in the Southern population, we can expect many phenotypes to have at least some genetic variance in this population.

## Discussion

Advances in genome sequencing have made it possible not only to expand the range of species for which reference genomes are available but also to sample the genetic diversity of individuals within the same species. Here we have explored individual genetic diversity in the model vertebrate, medaka (*Oryzias latipes*), by using inbred strains derived from various geographically distinct populations and from wild catches obtained from a single Southern Japanese population.

Our primary motivation for this study was to characterize a single medaka population from Southern Japan as a potential source for the first vertebrate near-isogenic wild panel. The sampled individuals are genetically highly diverse, as expected for a large population size. There is no discernible population structure in our sample, which is beneficial for mapping by association. Both medaka and human have substantially shorter LD than structured mouse populations. In the past, longer LD profiles were generally desirable in genetic panels for association analysis because this reduced the number of markers that needed to be typed to detect association. However, in the modern setting of inexpensive complete sequencing, an LD profile extending for shorter distances allows higher resolution and finer mapping of causal variants. This relatively tight LD will prove invaluable for the isolation of causative variants with 85% of SNP pairs in LD (from this limited sample set) mapping to single genes and 37% mapping to single exons. A larger panel will sample more polymorphic sites, and so we can expect this panel to nearly always have resolution to a single gene, and often to a single exon or regulatory region. As expected, the sample possesses considerable phenotypic diversity, with the phenotypic differences similar in magnitude to differences between inbred mouse strains.

During characterization of the progenitors of the panel, we also determined the sequence of a number of existing isogenic medaka strains sampled from several wild populations. Our additional deep sequencing data provide an estimate of the original reference sequence error rate at around 2 × 10^−5^ assuming that there has been no sequence divergence in this strain since 2002. This is better than the estimated error rates for draft quality genome sequencing. We are in the process of generating an improved reference assembly using the additional sequence generated in this project and other sources in the future. All the sequence data are freely available, and the differences identified have been submitted to the Single Nucleotide Polymorphism Database (dbSNP) and will be available on the Ensembl browser and other resources in the future. The list of variants provides a resource for current medaka researchers, in particular those aiming to map and understand the different phenotypes present in these strains. As expected, among the wild catches and the inbred lines there are 2235 nonsense mutations representing natural knockouts of particular protein coding genes.

These initial data have allowed us to further explore medaka population genetics. We lend genetic support to the current hypothesis that the Northern medaka population is very likely to be a distinct species that has not significantly interbred with the Southern medaka population since the Southern/Northern population split. The population history of Southern medaka suggests a recent bottleneck from a substantially larger population approximately 10,000 years ago. Two events present themselves as possible explanations for this bottleneck. First, it is tempting to speculate that this might be associated with the development of rice cultivation on the Japanese islands. Second, there may have been transgression of medaka populations after the last glacial age. 18,000 years ago, the sea level was about 120 m lower than at present, and Japan was directly connected to the Asian continent. Around 10,000 years ago, the sea level rose and the present plain fields were beneath sea level. To investigate this issue further, we need to contrast the Southern Japanese medaka population history to other histories from other geographic locations. The identification of three genes with putative amino acid changes due to positive selection is the start of exploring the impact of positive selection in medaka. The *thioredoxin reductase-3*, *kelch-like 10*, and *solute carrier family 41* (*slc41a3*) genes, involved in counteracting oxidative stress within the cell, spermatogenesis, and magnesium transport, respectively, might play fundamental roles for fitness and reproductive success. Intronic mutations also were found in other genes associated with membrane transport (*rims1*, *slc39a10*) and carbohydrate metabolism (*chst13*), as well as signaling (*wnt4a*) and chromatin (*histone 2ay*), suggesting that they may well be under noncoding, potentially regulatory positive selection, consistent with the evidence from stickleback (Jones *et al.* 2012).

Overall our study shows that a near-isogenic wild medaka panel has excellent potential for exploring vertebrate biology, analogous to the role of the Arabidopsis and Drosophila panels for plants and invertebrates, respectively. At the time of writing we have initiated the fourth generation of an inbreeding program for 200 independent medaka lines derived from this Kiyosu population. On completion of this program, these lines will be made broadly available to the community, with all data freely distributed. Of interest is that medaka can be housed in similar facilities to zebrafish, and the same phenotyping protocols can be applied to both fish species. Progress of this project can be tracked at http://www.ebi.ac.uk/birney-srv/medaka-ref-panel/, and we welcome participants both from established medaka and teleost laboratories and from the statistical genetics community.

## Supplementary Material

Supporting Information

## References

[bib1] Aida T., 1921 On the inheritance of color in a fresh-water fish, APLOCHEILUS LATIPES Temmick and Schlegel, with special reference to sex-linked inheritance. Genetics 6: 554–573 17245975 10.1093/genetics/6.6.554 PMC1200522

[bib2] Asai T. Senou H. Hosoya K., 2011 *Oryzias sakaizumii*, a new ricefish from northern Japan (Teleostei: Adrianichthyidae). Ichthyol. Explor. Freshwat. 22: 289–299

[bib3] Atwell S. Huang Y. S. Vilhjalmsson B. J. Willems G. Horton M., 2010 Genome-wide association study of 107 phenotypes in *Arabidopsis thaliana* inbred lines. Nature 465: 627–631 20336072 10.1038/nature08800 PMC3023908

[bib4] Bailey D. W., 1971 Recombinant-inbred strains. An aid to finding identity, linkage, and function of histocompatibility and other genes. Transplantation 11: 325–327 5558564 10.1097/00007890-197103000-00013

[bib5] Barrett J. C. Fry B. Maller J. Daly M. J., 2005 Haploview: analysis and visualization of LD and haplotype maps. Bioinformatics 21: 263–265 15297300 10.1093/bioinformatics/bth457

[bib6] Bloom J. S. Ehrenreich I. M. Loo W. T. Lite T. L. Kruglyak L., 2013 Finding the sources of missing heritability in a yeast cross. Nature 494: 234–237 23376951 10.1038/nature11867 PMC4001867

[bib7] Boc A. Diallo D. A. Makarenkov V., 2012 T-REX: a web server for inferring, validating and visualizing phylogenetic trees and networks. Nucleic Acids Res. 40: W573–579 22675075 10.1093/nar/gks485 PMC3394261

[bib8] Chen W. Li B. Zeng Z. Sanna S. Sidore C., 2013 Genotype calling and haplotyping in parent-offspring trios. Genome Res. 23: 142–151 23064751 10.1101/gr.142455.112 PMC3530674

[bib9] Collaborative Cross Consortium, 2012 The genome architecture of the Collaborative Cross mouse genetic reference population. Genetics 190: 389–401 22345608 10.1534/genetics.111.132639 PMC3276630

[bib10] Danecek P. Auton A. Abecasis G. Albers C. A. Banks E., 2011 The variant call format and VCFtools. Bioinformatics 27: 2156–2158 21653522 10.1093/bioinformatics/btr330 PMC3137218

[bib11] Drmanac R. Sparks A. B. Callow M. J. Halpern A. L. Burns N. L., 2010 Human genome sequencing using unchained base reads on self-assembling DNA nanoarrays. Science 327: 78–81 19892942 10.1126/science.1181498

[bib12] Durand E. Y. Patterson N. Reich D. Slatkin M., 2011 Testing for ancient admixture between closely related populations. Mol. Biol. Evol. 28: 2239–2252 21325092 10.1093/molbev/msr048 PMC3144383

[bib13] ENCODE Project Consortium Bernstein B. E. Birney E. Dunham I. Green E. D. Gunter C., 2012 An integrated encyclopedia of DNA elements in the human genome. Nature 489: 57–74 22955616 10.1038/nature11247 PMC3439153

[bib14] Flint J. Mackay T. F., 2009 Genetic architecture of quantitative traits in mice, flies, and humans. Genome Res. 19: 723–733 19411597 10.1101/gr.086660.108 PMC3647534

[bib15] Fu Y. Wen T. J. Ronin Y. I. Chen H. D. Guo L., 2006 Genetic dissection of intermated recombinant inbred lines using a new genetic map of maize. Genetics 174: 1671–1683 16951074 10.1534/genetics.106.060376 PMC1667089

[bib16] Furutani-Seiki M. Sasado T. Morinaga C. Suwa H. Niwa K., 2004 A systematic genome-wide screen for mutations affecting organogenesis in Medaka, *Oryzias latipes*. Mech. Dev. 121: 647–658 15210174 10.1016/j.mod.2004.04.016

[bib17] Gabriel S. B. Schaffner S. F. Nguyen H. Moore J. M. Roy J., 2002 The structure of haplotype blocks in the human genome. Science 296: 2225–2229 12029063 10.1126/science.1069424

[bib18] Gan X. Stegle O. Behr J. Steffen J. G. Drewe P., 2011 Multiple reference genomes and transcriptomes for *Arabidopsis thaliana*. Nature 477: 419–423 21874022 10.1038/nature10414 PMC4856438

[bib19] Gautier M. Vitalis R., 2012 rehh: an R package to detect footprints of selection in genome-wide SNP data from haplotype structure. Bioinformatics 28: 1176–1177 22402612 10.1093/bioinformatics/bts115

[bib20] Green R. E. Krause J. Briggs A. W. Maricic T. Stenzel U., 2010 A draft sequence of the Neandertal genome. Science 328: 710–722 20448178 10.1126/science.1188021 PMC5100745

[bib21] Groenen M. A. Archibald A. L. Uenishi H. Tuggle C. K. Takeuchi Y., 2012 Analyses of pig genomes provide insight into porcine demography and evolution. Nature 491: 393–398 23151582 10.1038/nature11622 PMC3566564

[bib22] Hyodo-Taguchi Y., 1980 Establishment of inbred strains of the teleost *Oryzias latipes*. Zool. Mag. 89: 283–301

[bib23] Hyodo-Taguchi Y., 1990 Inbred strains of the Medaka and their characteristics, pp. 129-142 in Biology of the Medaka, edited by Egami N. Yamagami K. Shima A. Tokyo University Press, Tokyo (in Japanese)

[bib24] Ishiguro N. Inoshima Y. Sasaki M. Matsui A. Hongo H., 2012 mtDNA variation and human-mediated introgression of indigenous Sus populations on several Indonesian islands. Mammal Study 37: 1–10

[bib25] Ishikawa Y. Yoshimoto M. Yamamoto N. Ito H., 1999 Different brain morphologies from different genotypes in a single teleost species, the medaka (*Oryzias latipes*). Brain Behav. Evol. 53: 2–9 9858800 10.1159/000006577

[bib26] Jones F. C. Grabherr M. G. Chan Y. F. Russell P. Mauceli E., 2012 The genomic basis of adaptive evolution in threespine sticklebacks. Nature 484: 55–61 22481358 10.1038/nature10944 PMC3322419

[bib27] Kasahara M. Naruse K. Sasaki S. Nakatani Y. Qu W., 2007 The medaka draft genome and insights into vertebrate genome evolution. Nature 447: 714–719 17554307 10.1038/nature05846

[bib28] Katsumura T. Oda S. Mano S. Suguro N. Watanabe K., 2009 Genetic differentiation among local populations of medaka fish (*Oryzias latipes*) evaluated through grid- and deme-based sampling. Gene 443: 170–177 19397956 10.1016/j.gene.2009.04.017

[bib29] Kimura T. Shimada A. Sakai N. Mitani H. Naruse K., 2007 Genetic analysis of craniofacial traits in the medaka. Genetics 177: 2379–2388 18073435 10.1534/genetics.106.068460 PMC2219511

[bib30] King E. G. Merkes C. M. McNeil C. L. Hoofer S. R. Sen S., 2012 Genetic dissection of a model complex trait using the Drosophila Synthetic Population Resource. Genome Res. 22: 1558–1566 22496517 10.1101/gr.134031.111 PMC3409269

[bib31] Langmead B. Salzberg S. L., 2012 Fast gapped-read alignment with Bowtie 2. Nat. Methods 9: 357–359 22388286 10.1038/nmeth.1923 PMC3322381

[bib32] Li H. Durbin R., 2011 Inference of human population history from individual whole-genome sequences. Nature 475: 493–496 21753753 10.1038/nature10231 PMC3154645

[bib33] Li H. Handsaker B. Wysoker A. Fennell T. Ruan J., 2009 The Sequence Alignment/Map format and SAMtools. Bioinformatics 25: 2078–2079 19505943 10.1093/bioinformatics/btp352 PMC2723002

[bib34] Liti G. Carter D. M. Moses A. M. Warringer J. Parts L., 2009 Population genomics of domestic and wild yeasts. Nature 458: 337–341 19212322 10.1038/nature07743 PMC2659681

[bib35] Loosli F. Koster R. W. Carl M. Kuhnlein R. Henrich T., 2000 A genetic screen for mutations affecting embryonic development in medaka fish (*Oryzias latipes*). Mech. Dev. 97: 133–139 11025214 10.1016/s0925-4773(00)00406-8

[bib36] MacArthur D. G. Balasubramanian S. Frankish A. Huang N. Morris J., 2012 A systematic survey of loss-of-function variants in human protein-coding genes. Science 335: 823–828 22344438 10.1126/science.1215040 PMC3299548

[bib37] Mackay T. F. Richards S. Stone E. A. Barbadilla A. Ayroles J. F., 2012 The Drosophila melanogaster Genetic Reference Panel. Nature 482: 173–178 22318601 10.1038/nature10811 PMC3683990

[bib38] Meyer M. Kircher M. Gansauge M. T. Li H. Racimo F., 2012 A high-coverage genome sequence from an archaic Denisovan individual. Science 338: 222–226 22936568 10.1126/science.1224344 PMC3617501

[bib39] Mongin E. Auer T. O. Bourrat F. Gruhl F. Dewar K., 2011 Combining computational prediction of cis-regulatory elements with a new enhancer assay to efficiently label neuronal structures in the medaka fish. PLoS One 6: e19747 21637758 10.1371/journal.pone.0019747 PMC3103512

[bib40] Peirce J. L. Lu L. Gu J. Silver L. M. Williams R. W., 2004 A new set of BXD recombinant inbred lines from advanced intercross populations in mice. BMC Genet. 5: 7 15117419 10.1186/1471-2156-5-7 PMC420238

[bib41] Pravenec M. Klir P. Kren V. Zicha J. Kunes J., 1989 An analysis of spontaneous hypertension in spontaneously hypertensive rats by means of new recombinant inbred strains. J. Hypertens. 7: 217–221 2708818

[bib42] Rembold M. Lahiri K. Foulkes N. S. Wittbrodt J., 2006 Transgenesis in fish: efficient selection of transgenic fish by co-injection with a fluorescent reporter construct. Nat. Protoc. 1: 1133–1139 17406394 10.1038/nprot.2006.165

[bib43] Retief J. D., 2000 Phylogenetic analysis using PHYLIP. Methods Mol. Biol. 132: 243–258 10547839 10.1385/1-59259-192-2:243

[bib44] Sabeti P. C. Reich D. E. Higgins J. M. Levine H. Z. Richter D. J., 2002 Detecting recent positive selection in the human genome from haplotype structure. Nature 419: 832–837 12397357 10.1038/nature01140

[bib45] Sasado T. Tanaka M. Kobayashi K. Sato T. Sakaizumi M., 2010 The National BioResource Project Medaka (NBRP Medaka): an integrated bioresource for biological and biomedical sciences. Exp. Anim. 59: 13–23 20224166 10.1538/expanim.59.13

[bib46] Sasaki S. Mello C. C. Shimada A. Nakatani Y. Hashimoto S., 2009 Chromatin-associated periodicity in genetic variation downstream of transcriptional start sites. Science 323: 401–404 19074313 10.1126/science.1163183 PMC2757552

[bib47] Setiamarga D. H. Miya M. Yamanoue Y. Azuma Y. Inoue J. G., 2009 Divergence time of the two regional medaka populations in Japan as a new time scale for comparative genomics of vertebrates. Biol. Lett. 5: 812–816 19586967 10.1098/rsbl.2009.0419 PMC2827986

[bib48] Sharopova N. McMullen M. D. Schultz L. Schroeder S. Sanchez-Villeda H., 2002 Development and mapping of SSR markers for maize. Plant Mol. Biol. 48: 463–481 12004892 10.1023/a:1014868625533

[bib49] Struyf A. Hubert M. Rousseeuw P. J., 1997 Integrating robust clustering techniques in S-PLUS. Comput. Stat. Data Anal. 26: 17–37

[bib50] Takeda H. Shimada A., 2010 The art of medaka genetics and genomics: what makes them so unique? Annu. Rev. Genet. 44: 217–241 20731603 10.1146/annurev-genet-051710-151001

[bib51] Takehana Y. Nagai N. Matsuda M. Tsuchiya K. Sakaizumi M., 2003 Geographic variation and diversity of the cytochrome b gene in Japanese wild populations of medaka, *Oryzias latipes*. Zoolog. Sci. 20: 1279–1291 14569151 10.2108/zsj.20.1279

[bib52] Takehana Y. Jeon S. R. Sakaizumi M., 2004 Genetic structure of Korean wild populations of the Medaka Oryzias latipes inferred from allozymic variation. Zoolog. Sci. 21: 977–988 15459457 10.2108/zsj.21.977

[bib53] Toyama K., 1916 On some Mendelian characters. Rep. Jap. Breed. Soc. 1: 1–9 (in Japanese)

[bib54] Untergasser A. Nijveen H. Rao X. Bisseling T. Geurts R., 2007 Primer3Plus, an enhanced web interface to Primer3. Nucleic Acids Res. 35: W71–74 17485472 10.1093/nar/gkm306 PMC1933133

[bib55] Voight B. F. Kudaravalli S. Wen X. Pritchard J. K., 2006 A map of recent positive selection in the human genome. PLoS Biol. 4: e72 16494531 10.1371/journal.pbio.0040072 PMC1382018

[bib56] Wittbrodt J. Shima A. Schartl M., 2002 Medaka—a model organism from the far East. Nat. Rev. Genet. 3: 53–64 11823791 10.1038/nrg704

[bib58] Yamamoto T-O., 1953 Artificially induced sex-reversal in genotypic males of the medaka (*Oryzias Latipes*). J Exp Zool 123: 571–594

[bib57] Zhao S. Zheng P. Dong S. Zhan X. Wu Q., 2013 Whole-genome sequencing of giant pandas provides insights into demographic history and local adaptation. Nat. Genet. 45: 67–71 23242367 10.1038/ng.2494

