# Supplementary material for: Genomic and Phenotypic Characterization of a Wild Medaka Population: Towards the Establishment of an Isogenic Population Genetic Resource in Fish
Source: G3 (Bethesda). 2014 Jan 9;4(3):433–45. doi: 10.1534/g3.113.008722 (PMC3962483; doi:10.1534/g3.113.008722)
Supplement: Supporting Information [file supp_4_3_433__index.html]

Genomic and Phenotypic Characterization of a Wild Medaka Population: Towards the Establishment of an Isogenic Population Genetic Resource in Fish — Supporting Information 

# Genomic and Phenotypic Characterization of a Wild Medaka Population: Towards the Establishment of an Isogenic Population Genetic Resource in Fish

## Supporting Information for Spivakov *et al.*, 2014

**Files in this Data Supplement:**

- Supporting Information - Figures S1-S3 and Tables S1-S6 (PDF, 891 KB)
- Figure S1 - Boxplots of measured morhpometric features in the different inbred strains. (PDF, 403 KB)
- Figure S2 - Morphometric analysis of four inbred Southern lines, two inbred Northern lines and two trios from the Kiyosu wild population: Dorsal features. (PDF, 463 KB)
- Figure S3 - Morphometric analysis of four inbred Southern lines, two inbred Northern lines and two trios from the Kiyosu wild population: Lateral features. (PDF, 450 KB)
- Table S1 - Microsatellite markers used. (PDF, 405 KB)
- Table S2 - Microsatellite alleles detected in wild Kiyosu Population. (PDF, 505 KB)
- Table S3 - Introgression analysis. (PDF, 403 KB)
- Table S4 - iHS analysis (PDF, 399 KB)
- Table S5 - Primers designed for SNP verification sequencing. (PDF, 433 KB)
- Table S6 - Details of sequence coverage for the additional inbred medaka lines. (PDF, 400 KB)
