# Supplementary material for: Genomic and Phenotypic Characterization of a Wild Medaka Population: Towards the Establishment of an Isogenic Population Genetic Resource in Fish
Source: G3 (Bethesda). 2014 Jan 9;4(3):433–45. doi: 10.1534/g3.113.008722 (PMC3962483; doi:10.1534/g3.113.008722)
Supplement: Supporting Information [file supp_g3.113.008722_008722SI.pdf]

Genomic and phenotypic characterization of a wild medaka population: Towards the establishment of an isogenic population genetic resource in fish

Mikhail Spivakov[1,6]*, Thomas O. Auer[2,7]*, Ravindra Peravali[3], Ian Dunham[1], Dirk Dolle[1,2], Asao Fujiyama[4], Atsushi Toyoda[4], Tomoyuki Aizu[4], Yohei Minakuchi[4], Felix Loosli[3§], Kiyoshi Naruse[5§], Ewan Birney[1§], Joachim Wittbrodt[2§]

[1]European Bioinformatics Institute (EMBL‐ EBI), Wellcome Trust Genome Campus, Hinxton, Cambridgeshire, UK

[2]Centre for Organismal Studies, Heidelberg University, Germany

[3]Karslruhe Institute of Technology, KIT, Karlsruhe, Germany

[4]Comparative Genomics Laboratory, Center for Information Biology, National Institute of Genetics, Shizuoka, Japan

[5]National Institute for Basic Biology, NIBB, Laboratory of Bioresources, Okazaki, Japan

[6]Present address: Babraham Institute, Cambridge, UK

[7]Present address: Neuronal Circuit Development Group, Unité de Génétique et Biologie du Développement, U934 / UMR3215, Institut Curie, Paris, France

*These authors contributed equally to this work

§Corresponding authors

Email addresses:

MS: Mikhail.Spivakov@babraham.ac.uk, TOA: thomas.auer@curie.fr, RP: ravindra.peravali@kit.edu, ID: dunham@ebi.ac.uk, DD: ddolle@ebi.ac.uk, AF: afujiyam@gmail.com, AT: atoyoda@nig.ac.jp, TA : toaizu@nig.ac.jp, YM: yminakuc@nig.ac.jp, FL: felix.loosli@kit.edu, KN: naruse@nibb.ac.jp, EB: birney@ebi.ac.uk, JW: jochen.wittbrodt@cos.uni-heidelberg.de.

DOI: 10.1534/g3.113.008722

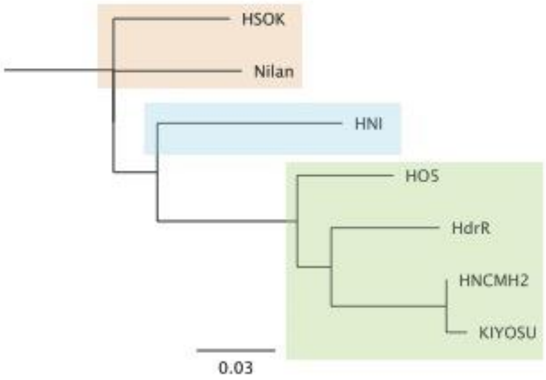

Figure S1 Boxplots of measured morhpometric features in the different inbred strains. A) Dorsal features. B) Lateral features. X-axis: Pixel values for each measurement. Grey shading: Southern inbred strains. Yellow shading: Northern inbred strains.

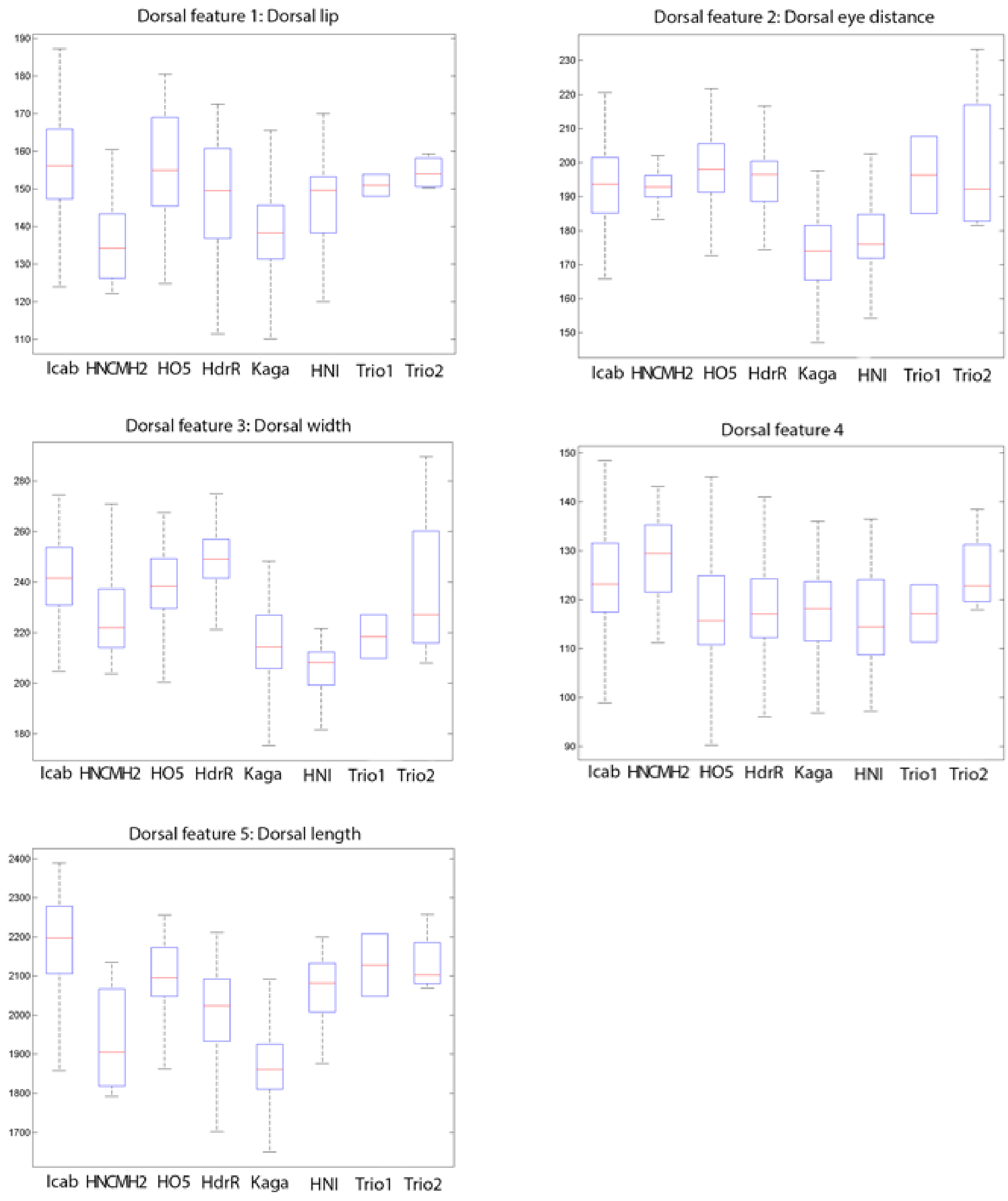

Figure S2 Morphometric analysis of four inbred Southern lines, two inbred Northern lines and two trios from the Kiyosu wild population: Dorsal features. The respective definitions of Features 1 to 5 are indicated in Figure 6A. A significant difference in all features between the six inbred lines is observed. The Kiyosu morphometric analysis is preliminary as the dataset has low sample numbers (N < 10), whereas for the inbred lines N > 75. The differences between the Kiyosu datasets to the other datasets are therefore not statistically significant.

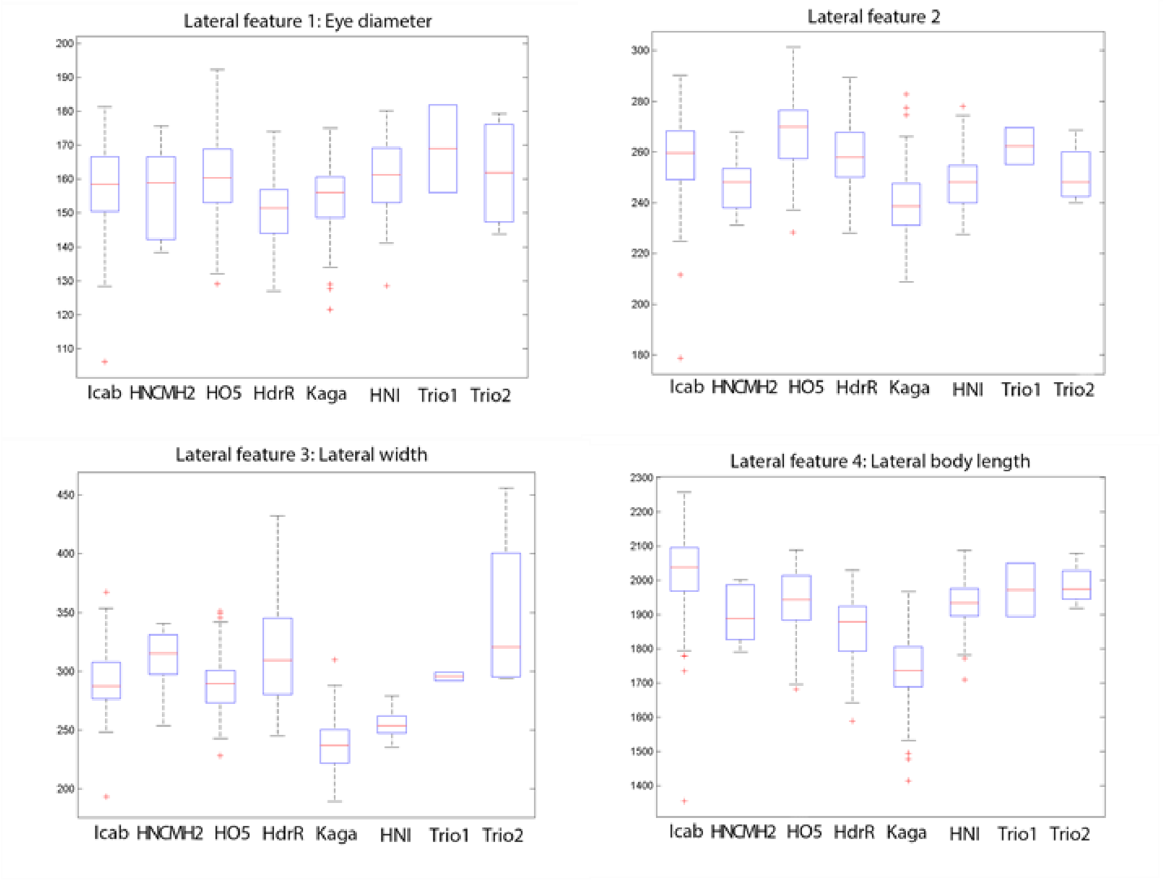

Figure S3 Morphometric analysis of four inbred Southern lines, two inbred Northern lines and two trios from the Kiyosu wild population: Lateral features. The respective definitions of Features 1 to 4 are indicated in Figure 6A. A significant difference in all features between the six inbred lines is observed. The Kiyosu morphometric analysis is preliminary as the dataset has low sample numbers (N < 10), whereas for the inbred lines N > 75. The differences between the Kiyosu datasets to the other datasets are therefore not statistically significant.

Table S1 Microsatellite markers used. Oligonucleotoide primer sequences, genomic locations and amplicon lengths for the microsatellite markers analyzed in the Kiyosu population sample.

Linkage Group	Forward Primer	Reverse Primer	Genomic location	Estimated amplicon size
LG1	AGCATTTCTAGTTTGCGTTT	CTAGTTTTTCCGGACAGTCA	1:24815630:24815850:1	202
LG2	ATATCGACAGGTATGGATGG	TCACATCTAGACCTGGAAGC	2:27141500:27141900:1	243
LG3	TTATGATCCCCATATTTGATT	AGAGGGACACTGGGACAG	3:9396729:9397030:1	301
LG4	GCTTTAAATTAGAGTTCATAATACTGC	GCTTTCATTAACATCAGGAAG	4:22460213:22460453:1	229
LG5	GTTTTTGAAAGTTTTATTTTTGG	TCAAAGCTAGTATTATGTAAACACAG	5:8443091:8443291:1	181
LG6	ATGTCCACCGACAAACAC	AGTAGATGCCCAGTGAGAGA	6:10841500:10841800:1	250
LG7	AGCTTGCCCTTTTTCTACAT	GTTTGGAGACCCACTGTTT	7:19386200:19386460:1	188
LG8	GCCAAAATGCCTTTTTATTT	TCCACATCAGAGCACATTC	8:21636852:21637100:1	209
LG9	TTTTCATCTGCTCCTGATTT	AGAGTTTCAGCAAGCAATGT	9:26192300:26192600:1	212
LG10	CACCAGCTGAGTTTGTAATG	CAAGAGAAGGGAAGTCGTG	10:10113640:10113860:1	208
LG11	ACAAGTATAGCTGCCCTCAG	CCTGCTTTCTTTTTGATTTT	11:4573925:4574200:1	205
LG12	GCTGTTCACAGCTGATGTC	ATATTGGCACAAGCTGTTTT	12:15240800:15241200:1	202
LG13	TTAGGCTCAATCTGTTATTTACAGATTAC	ATCCACAGGCTCAGCATCTT	13:10169674:10169904:1	231
LG14	TTGCTGTCCCTGTGTGTTTG	GCTTGAAGGCAACAGCTTTG	14:7011652:7011851:1	200
LG15	TGCCATAAATGAGGGAATTTAC	TCAGTGTGAATCAGTTTCCTGT	15:7515398:7515597:1	200
LG16	AATGACTTGGTGTCATATAATTCCA	AATTAAATATCATCTTGGCAACATTATA	16:7247259:7247458:1	200
LG17	GGATTGCTGCTGAGTGTAATTC	CAATTTCCAGTTTCACTGACTGG	17:8333779:8333969:1	191
LG18	AGCTTAGCAGACCGTTTTAGG	CCTGCCGTGGATCTCAG	18:5214059:5214245:1	187
LG19	GGTTTTTGCATCCTGCC	GCTACCGAGTCCTTTGAATAAAG	19:13774098:13774297:1	200
LG20	AAAAGCTGTATGAACAGATTTGC	TGAGAGCCTGTTGCCTTC	20:7665987:7666186:1	200
LG21	TAGCGGAGGCTGGCTG	CAGAGCGCGCGGAGC	21:10273689:10273888:1	200
LG22	AGAAAAGGAATGAGCCAGACC	GTGTTTGTGCCTGGAAATAGG	22:7909633:7909831:1	199
LG23	GTGGAGGCTGCCGACGG	CTCTGCTGTCTGCCTGCC	23:8615246:8615443:1	198
LG24	AGTTTTTGGCTACATTCATAGTCAG	GGAGAGGGGTTTCTAGGGA	24:9528803:9529032:1	230

Table S2 Microsatellite alleles detected in wild Kiyosu Population. Microsatellite alleles were tested in 109 fish from the wild Kiyosu Population. PCR amplification failed in 4 fish (samples 1, 5, 6, 7) giving allele results for 105 fish. For each microsatellite assay labeled as described in Supplementary Table 1, the two alleles identified in each assayed Kiyosu individual are listed.

Sample	D-loop Sequencing	LG3	LG3	LG6	LG6	LG13	LG13	LG14	LG14	LG16	LG16	LG17	LG17	LG18	LG18	LG22	LG22	LG24	LG24
1	C	-	-	-	-	-	-	-	-	-	-	-	-	-	-	-	-	-	-
2	G	L	M	C	H	A	A	A	A	A	A	C	C	B	B	K	K	A	B
3	H	E	E	F	I	D	A	F	K	A	A	C	C	B	B	O	O	A	E
4	H	U	U	-	-	G	A	A	B	A	A	C	F	B	B	A	A	B	D
5	A	-	-	-	-	-	-	-	-	-	-	-	-	-	-	-	-	-	-
6	H	-	-	-	-	-	-	-	-	-	-	-	-	-	-	-	-	-	-
7	H	-	-	-	-	-	-	-	-	-	-	-	-	-	-	-	-	-	-
8	C	Q	S	A	E	G	G	A	A	A	A	A	A	A	B	E	H	B	C
9	H	N	N	-	-	-	-	-	-	A	A	A	A	-	-	-	-	-	-
10	C	B	O	-	-	-	-	-	-	A	B	F	F	-	-	-	-	-	-
11	H	M	Q	-	-	-	-	-	-	A	A	B	C	-	-	-	-	-	-
12	C	E	L	-	-	-	-	-	-	A	A	C	G	-	-	-	-	-	-
13	H	F	M	A	E	A	F	A	N	A	A	B	D	B	B	F	N	A	C
14	G	B	L	A	G	A	A	A	K	A	A	A	D	B	B	D	H	A	A
15	A	C	J	E	F	D	E	A	G	A	A	C	F	A	B	B	E	A	D
16	H	H	M	A	A	J	K	D	L	A	A	B	C	B	B	E	I	C	K
17	H	M	M	D	E	A	A	B	C	A	A	C	E	B	B	E	E	B	C
18	E	A	L	A	E	A	D	B	B	A	A	B	E	B	B	J	J	A	D
19	D	E	I	F	G	A	D	O	P	A	A	C	D	B	B	B	C	B	D
20	H	F	K	A	E	A	I	A	M	A	A	C	E	B	C	E	E	C	E
21	H	Q	Q	A	A	A	B	G	G	A	A	C	F	A	B	A	J	D	G
22	C	K	S	E	I	D	I	A	J	A	A	D	F	A	B	E	J	D	D
23	C	M	M	A	I	D	H	A	G	A	A	A	A	B	B	G	G	C	E
24	H	K	N	D	I	A	C	C	D	A	A	B	C	B	B	B	C	A	C
25	H	A	J	E	F	A	A	C	H	A	A	D	F	B	B	C	H	A	C
26	C	U	U	D	E	A	E	A	A	A	A	A	D	B	B	C	E	C	E
27	-	G	I	E	G	A	C	B	C	A	A	A	E	B	B	F	M	A	L
28	C	H	O	B	E	D	F	B	B	A	A	C	D	B	B	A	A	A	C

29	C	F	I	D	E	A	A	-	-	A	A	B	E	B	B	E	L	-	-
30	-	A	I	A	E	D	D	A	A	A	A	A	D	B	B	C	C	A	D
31	G	K	R	A	H	-	-	-	-	A	A	B	D	B	B	G	I	-	-
32	C	B	P	E	G	A	A	A	C	A	A	B	C	B	C	A	E	A	B
33	-	I	K	A	H	B	B	D	D	A	A	C	E	A	B	C	J	F	F
34	G	E	E	A	G	A	E	I	O	A	A	B	C	A	B	I	I	B	B
35	B	D	L	A	G	D	D	B	I	A	A	A	D	B	B	A	E	B	C
36	G	P	P	B	F	A	B	B	C	A	A	C	D	B	B	C	G	A	D
37	C	K	Q	E	G	B	D	A	M	A	A	B	D	A	B	C	N	A	E
38	C	D	G	A	G	A	C	B	G	A	A	C	D	B	B	E	E	A	A
39	C	E	J	A	C	D	F	C	J	A	A	A	F	A	B	C	H	C	E
40	H	L	O	A	F	C	D	D	F	A	A	B	D	A	B	C	E	A	C
41	G	E	O	A	A	E	H	A	B	A	A	B	D	B	B	K	K	A	E
42	C	D	J	E	G	A	C	B	M	A	B	C	H	A	B	E	M	B	C
43	H	R	R	C	G	B	B	I	O	C	D	C	C	A	B	E	E	F	J
44	H	C	P	F	I	A	H	D	K	A	A	D	D	A	B	E	P	E	E
45	H	-	-	F	G	B	B	B	B	A	B	A	G	A	B	C	E	F	F
46	C	A	S	A	I	A	C	A	A	A	A	A	G	B	B	N	N	C	C
47	C	L	M	E	G	C	G	C	H	A	A	A	E	B	B	A	E	C	C
48	H	E	R	A	E	A	D	B	F	A	A	C	D	B	B	C	G	A	D
49	H	I	L	-	-	-	-	-	-	A	A	B	C	-	-	-	-	-	-
50	C	-	-	-	-	-	-	-	-	A	A	B	B	-	-	-	-	-	-
51	E	F	G	B	E	A	D	A	M	A	A	A	A	A	B	A	H	A	D
52	H	G	L	E	E	A	C	F	F	A	A	A	J	B	B	C	C	A	A
53	H	A	G	A	D	F	I	B	I	A	A	C	E	B	B	H	I	A	D
54	C	B	K	F	F	E	F	A	A	A	A	C	C	B	B	E	O	A	E
55	H	O	Q	A	E	A	D	F	I	A	A	C	D	B	B	E	O	A	C
56	H	E	Q	F	J	A	A	G	I	A	A	B	B	B	B	F	L	B	C
57	H	M	P	A	E	E	G	E	J	A	A	B	C	B	B	C	G	A	C
58	C	A	L	A	A	C	H	A	B	A	A	A	A	B	B	B	E	C	E
59	H	L	O	D	G	D	E	N	N	A	A	A	A	B	B	C	O	A	C
60	B	H	O	A	A	A	C	H	N	A	A	A	F	B	B	D	L	A	B

61	H	M	S	A	H	D	D	C	J	A	A	C	F	B	B	B	O	B	D
62	C	H	Q	G	I	B	B	M	M	A	A	A	A	B	B	B	J	F	I
63	D	K	S	A	F	A	D	K	M	A	A	B	B	B	B	E	E	A	G
64	H	C	D	A	D	B	F	D	N	A	A	B	C	B	B	B	N	B	C
65	H	F	K	A	A	B	C	A	C	A	A	A	A	B	B	A	A	C	D
66	H	E	H	A	E	A	A	M	N	A	A	A	D	B	B	C	L	A	C
67	H	E	Q	A	E	A	D	A	L	A	A	B	B	A	B	B	B	A	C
68	H	K	O	D	E	A	A	A	I	A	A	E	E	B	B	E	I	E	E
69	H	E	F	E	G	A	C	A	B	A	A	B	B	B	B	N	P	B	F
70	C	P	S	A	G	C	D	B	K	A	A	B	B	B	B	D	J	B	C
71	H	K	N	E	G	C	D	A	B	A	A	A	J	B	B	G	L	C	D
72	C	E	M	C	G	C	D	G	N	A	A	A	F	B	B	-	-	A	C
73	G	K	P	G	G	D	H	C	O	A	A	A	A	B	B	B	E	A	B
74	H	D	K	A	A	A	D	B	B	A	A	C	F	B	B	C	M	A	B
75	D	E	H	E	G	A	C	A	C	A	A	B	B	B	B	C	K	C	C
76	C	A	L	A	F	A	C	A	C	A	A	C	E	B	B	B	C	B	D
77	C	N	Q	A	A	A	F	A	A	A	A	A	D	B	B	A	A	A	C
78	H	E	M	A	I	A	C	A	A	A	A	A	D	A	B	F	I	A	D
79	H	B	T	-	-	D	H	I	J	A	A	C	C	-	-	-	-	B	C
80	H	A	E	G	H	A	C	F	J	A	A	C	D	B	B	C	N	B	B
81	D	F	L	A	J	C	C	E	H	A	A	B	C	B	B	B	E	A	C
82	H	M	M	A	I	A	C	B	P	A	A	B	D	B	B	E	E	B	C
83	H	E	K	A	F	A	H	A	O	A	A	A	D	A	B	B	L	B	B
84	D	E	M	A	E	A	A	A	A	A	A	A	D	B	B	B	E	C	C
85	C	A	G	E	E	A	A	H	I	A	A	B	C	A	B	J	O	A	D
86	G	E	M	A	E	D	D	B	I	A	A	A	A	B	B	C	C	B	D
87	H	M	P	D	E	C	D	A	A	A	A	B	C	A	B	L	L	A	D
88	H	E	L	A	G	A	E	A	B	A	A	C	F	B	B	B	M	A	C
89	C	E	E	B	E	A	G	D	G	A	A	C	D	B	B	E	G	A	C
90	H	B	K	A	A	D	F	G	I	A	A	F	F	A	B	J	L	A	C
91	H	A	I	A	A	A	C	A	N	A	A	B	B	A	B	C	C	C	D
92	C	F	P	A	G	F	I	A	D	A	A	B	G	A	B	G	I	A	C

93	H	I	N	F	J	A	H	A	G	A	A	C	I	A	B	C	C	A	A
94	G	E	K	E	H	-	-	-	-	A	A	B	I	A	B	-	-	-	-
95	H	G	O	E	E	A	B	A	A	A	A	A	A	B	B	C	C	D	D
96	H	F	P	A	C	A	E	I	G	A	A	A	A	A	B	K	L	B	D
97	H	A	M	C	F	B	D	A	C	A	A	A	I	B	B	G	H	C	D
98	D	F	L	E	E	D	D	A	M	A	A	A	A	B	B	B	C	A	C
99	H	G	I	E	I	A	A	G	H	A	A	A	F	B	D	K	K	A	B
100	H	B	P	A	G	A	C	A	I	A	A	B	C	B	B	B	E	A	D
101	H	E	E	A	C	D	D	A	I	A	A	A	A	A	B	C	D	A	D
102	F	E	G	E	H	A	D	B	F	A	A	A	F	A	B	E	G	D	E
103	C	I	T	A	E	C	E	D	J	A	A	B	E	B	C	C	K	A	C
104	H	D	S	A	F	F	H	A	A	A	A	B	B	A	B	E	K	C	D
105	H	L	M	C	E	C	D	D	F	A	A	C	D	B	B	C	C	D	E
106	C	F	O	A	G	A	D	I	O	A	A	C	D	B	B	G	G	B	C
107	H	N	O	G	G	A	I	B	C	A	A	B	C	A	B	J	L	A	C
108	H	E	K	A	E	A	C	H	L	A	A	A	A	A	B	C	E	A	E
109	H	E	I	A	I	A	C	D	D	A	A	B	C	A	B	E	N	A	A

Table S3 Introgression analysis. Tests for introgression of the P3 strain into P1 or P2 strains that are more closely related with each other than to P1, using stickleback as the outgroup (O). D% is the introgression test statistic, on which the standard error (SE), 95% confidence intervals (CI) and Z-scores were estimated using block-wise jackknife (Durand *et al.* 2011).

P1	P2	P3	O	D%	SE	95% CI	Z-score
**Kaga**	**Hni**	**HdrR**	Stickleback	**3.6**	**1.3**	**2.5**	**2.77**
Kaga	Hni	Wild Southern	"	2.6	1	2	2.5
Kaga	Hni	Wild Southern (SNPs with HdrR)	"	-4	3.6	7.1	1.1
**Nilan**	**Hsok**	**HdrR**	**"**	**–12.3**	**4.66**	**9.1**	**2.64**
Nilan	Hsok	Wild Southern	"	–10.4	5.27	10.3	1.96
Nilan	Hsok	Wild Southern (SNPs with HdrR)	"	7.6	6.5	12.8	0.6
Nilan	Hsok	Kaga	"	–0.7	2.5	4.9	0.28

Tests for introgression of the P3 strain into P1 or P2 strains that are more closely related with each other than to P1, using stickleback as the outgroup (O). D% is the introgression test statistic, on which the standard error (SE), 95% confidence intervals (CI) and Z-scores were estimated using block-wise jackknife.

Table S4 iHS analysis. Numbers of SNPs showing evidence of recent positive selection in regions with different levels of evolutionary conservation versus those expected at random. Expected values, their standard deviations and p-values were estimated from permutation tests.

Type	Observed	Expected	Standard deviation (+/-)	Fold change	Direction of difference	P
Non-conserved	87	60	7	1.45	Enriched	<0.005
At least 1 other teleost	105	131	7	0.80	Depleted	<0.005
At least 2 other teleosts	95	110	7	0.86	"	0.014
At least 3 other teleosts	72	89	7	0.81	"	0.008
Four other teleosts	38	27	5	1.41	Enriched	0.032

Numbers of SNPs showing evidence of recent positive selection in regions with different levels of evolutionary conservation versus those expected at random. Expected values, their standard deviations and p-values were estimated from permutation tests.

Table S5 Primers designed for SNP verification sequencing. PCR amplification primers are given for the SNP resequencing amplicons. For the coding SNPs, the original reference sequence indicated a coding triplet, whereas a stop codon was indicated by our height throughput sequencing. The status column indicates whether PCR amplification and sequencing confirmed the new sequence.

NONSENSE SNPs

Strain	Chromosome	Position	Reference triplet	Detected triplet	Reference amino acid	Forward primer	Reverse primer
HNI	chr23	20491840	TTA	TAA	L	TCAGTGGCTGTAAAAGGCTGTAGATTG	ACCACACAGATGCGAAAAACACATCC
HNI	chr8	1955292	CGA	TGA	R	ACCAGAATCCTTCTCCTCCACCTGC	CAAGCTGTGTAAGTGGGCCTGAGTC
HNI	chr7	1468063	TGG	TAG	W	AGTGTCCCATTTTGCCTTCAACAGC	CGGTTGCTTCTGCAAAGCCTATTCTCC
HNI	chr18	2964751	AAG	TAG	K	CTTTGCGTAGAATCTGATGGGACAGC	TGTATGCCTGCCTTTTCAATCAGCG
HNI	chr2	20573966	CAG	TAG	Q	CCGAGGTAAATTCTTTTCTGGTCAGTGG	GGGTTGTTTGTGCTTGGCTTCCCTG
HNI	chr14	15525786	TAC	TAA	Y	ACAACGACGTGAATATTCTGGAGGGG	AAGTCGCTTTTTACTGCGCACCTGG
Kaga	chr3	2245314	TCA	TGA	S	TCTTGCAGCTGGGGGAGGTGATGG	AGTTCCTCGTCTGCAATTGGTTGGC
Kaga	chr5	14257733	CAA	TAA	Q	TCAATTATTATCGTGGGATGTACTGGTGC	TGTGGATGGATGGAACAAGCTCAACG
Kaga	chr14	7969562	CAA	TAA	Q	AGCTCAAGGTGGTGTAGGTTCCAGG	CTCGTGGGTCTCCCGTTCTCTCATC
Kaga	chr13	5610005	TCG	TAG	S	CGCCAGATCAAAGATAGGGGGAAAGC	TATGACGTTCACACTGGAGAAGGGGC
Nilan	chr8	22537356	CGA	TGA	R	CCACAGAGCCACAGGAGATGTGATG	AGAATCCTAAAAGCAGCAGACGCCG
Nilan	chr22	1757977	GGA	TGA	G	TCGTTTCCTGCAGATCCTGAGGTGG	GGGTCAGGGTTTTAGCCGTTTCTGC
Nilan	chr16	6784490	TGT	TGA	C	TCACTTTTGCGTCTCTGTCTTTAGGTGG	TGTCTGACAGCTTAACAAGGATCTGTTTG
Nilan	chr7	4911752	TGG	TAG	W	TCTGCTGTACGCAGCGGACTTTTTC	AACTTTAAGCTTTTCCTGGCGCAGC
Nilan	chr18	17668284	AAA	TAA	K	ACACACCTTTCTTGACTTCAGCCGC	AACGAGAAGACAGAAGACGGTGAGGC
Nilan	chr15	11785459	GAG	TAG	E	ACATTTCTGACTAATTTCAGCAGCAGGTC	CGTAGTTGGACTTTATGGAAGCCAGG

NON-CODING SNPs (Kaga)

Strain	Chromosome	Position	Reference base	Detected base	Forward primer	Reverse primer
Kaga	chr3	8401434	T	G	AGGTTTGTTTACCAGTGATTGTGACTGAG	GGTGCGTTATGTAGCTGCTGTTCGG
Kaga	chr4	31533375	G	T	AGCAGCCAGTGCCAAGAAAACAGAG	TTTCAATGGCCCTTTAGGGGCTCCG
Kaga	chr6	15965403	G	C	TGCACGATGTTGCAAAATATTACCCCC	CGTCTTGGTCACAGCGAACTCTAACG

Kaga	chr7	27519929	G	A	TGCACTCTTGCCATAAACAGTCATCC	TGTCTGTCTGGAAGCAGCATAACCC
Kaga	chr19	19825516	A	T	CCTGCACAAAACCCACACATACACG	AGGCAGAGTTTGACGTGGATCAAGC
Kaga	chr22	5161675	G	A	GACCTTTTAACTGGGGCCCTGTGTG	TCTCCTCCCCCTTTGTTGTGTGCTG
Kaga	chr20	9670880	A	T	GCAAGCTGCCCTAACCAATGATCCG	CTCACATGCCTAGCAGCAGTGACAG
Kaga	chr22	24304734	T	C	TGGGGTTCTAGTGAAGGAAGGCTTGG	GCCTCTTTTAGCCGTCCTCCTGTTG
Kaga	chr5	28448196	A	T	CTGATGTGACACACTCCAAAAACAGGC	ACGAGCCTGTTGGGAGTCAAAAAGC
Kaga	chr17	2618509	C	G	GCTGCTTCTAAGGTCAGTCCGTTTG	GACAACTAAAAGCAGCAAGCGCAGC

RANDOM SNPs (HdrR)

Strain	Chromosome	Position	Reference base	Detected base	Forward primer	Reverse primer
HdrR	chr1	16324653	C	A	CAAGGGAGGCACCAATGAACCTGAG	TTTTGATGTGGAGGAACAGTAGGCTG
HdrR	chr1	33512684	T	G	TGTTTGTTGCAGAGCGAAGCAGCAG	GACCGTGAATGTAAAAGGGAGCCCG
HdrR	chr9	31027771	T	G	GCACCAGGCTTGTCCACAGTTAAGG	AAGACATCCGTGTCCGTTCCAGTGC
HdrR	chr16	3550201	C	A	AGCCGACGTGGCCTTTGTGTGGG	TCTTCTGGTGGTTTCTACCCTCTGGG
HdrR	chr1	27318612	A	G	CAAGCAGGTTTTGCTTTCAACTCCGC	ACCTGCCAAAAGTGAAACCTAACGGC
HdrR	chr16	5495844	A	G	GCAACCATGGCAGTTGCACCATAAAC	TGAGCGCTCTTCCTCTCATCCATGC
HdrR	chr1	23401471	C	T	CCGATACGCTGGTTTCTACGGTCTG	TGTCTGTGTTTGAACTGTGTCTCCAGC
HdrR	chr1	15819568	A	G	AAAGAGTCAGATGGAAGAGCTGAAAGATG	TGATTGCGTGTACTCCTCCTCCAAG
HdrR	chr1	19723612	C	T	ACATGGTACTCACAACGCTCATTGGC	CACAGAAGTACCGCTGAAGGTGCTG
HdrR	chr22	21769957	T	C	GTGAGAGCGCTGGAGATGAGAGGAG	CTGGAAAACACATTTCACGCTGCGG
HdrR	chr8	2893859	A	G	ACAAGTTGGGCATATCCACAGGACG	GTTTACCACACCGATAATTTTAAC
HdrR	chr16	20019740	T	C	GGGACGCGAAGGTGAGCAATGAATC	TCTGGCAGAGACCCACCAGCTTAC
HdrR	chr8	14657015	C	T	CATGCCTGTGTGCTTGACTATTGCAG	CCGGTTCAAGAAGTCGTTAAACTGG
HdrR	chr13	5993620	T	A	CGTTTTTTAAGGAAAACCTTGGATGG	TGGACTGAGTGACCCCTAACCCCTC
HdrR	chr9	11937557	T	C	TCCTAACAGCAAAGGACTCAAAACCC	GTGACACAGGGCTGTGCATAGAGAG
HdrR	chr24	17741133	G	T	GTCCACCTTTTTCAGAGCTCTTTCTGAC	TCAGGCCTTCTTTGTCACTGCCTGG
HdrR	chr2	15141629	G	A	ATGTCCCCCTAAAATCCCTCTAGGC	CATTGCTGCAGTCCGGAGTCAAAGG
HdrR	chr22	21202891	A	G	TAGGTGTGAGTGCGGGAGTGAATGG	TCCAGGTCCAGGTCCAGGTCTATCC
HdrR	chr12	5608651	A	G	CTGCTGCATTTAAACATCTGACTTTCAGG	TACATGCAGGCAAACACAATGAAAATGTC
HdrR	chr9	15611521	C	A	GCATGTGTGACCCTGTCCGTGTAAG	GCTTATCAACATGCCAATGTCCCCC

HdrR	chr22	21589634	A	G	AAATGCAGGGTTGAACGCTCTAATGC	TCCAGGATTTCCAGGATTTTTGCCTC
HdrR	chr1	21776066	G	T	GAACCTGATCAGGAAGAAGTTAGGG	TGCCTGTTCTTTGAAACTGTGCCTC
HdrR	chr1	21103193	A	T	TCCAGCCCGCTCAGATGTTGTAGAC	GAATGGGGAGTCTGTATCGCCCCTG
HdrR	chr1	33292558	A	C	ACGGGCACGTGACTGGTGTCTATG	GGATACCGTGTGACGGTGCGTAATAG
HdrR	chr10	22338538	C	A	AACCCCAACAACCCTTTTGGAGTGC	TGAGGCGACTCAGCCTTACAGCTTG
HdrR	chr1	29019329	G	C	CGCCTTGGTCTGCATCAAGAGTGAC	TGCGCTCCTAAGCTTTCTCTCAGCG
HdrR	chr1	28914573	C	G	AGCAGGCACTGTGAAGAATACCACG	ACTTCAAGGCTCACTTTTTGGTGCC
HdrR	chr13	21682092	A	G	TGTTTTACCCTTAAACAATGGTTTGCTGC	ATGGAAATCTGCACAGGTGCAAGCC
HdrR	chr15	27757265	A	C	CATCAAAACCTAAGACAACCTCCTGCC	TGTGACGTTTTTGTGTTTCTGTGAGAACG
HdrR	chr20	3264988	C	T	GAGGTGTGAACTGTCGTCTGAAGGC	AGGCAGTTTTATGTTTGAGCGCCCC
HdrR	chr1	22961788	T	A	GCAGCCACTGGTATTTTGTGGGGAC	CCTGGGGGACGTTCCCTCGATTAG
HdrR	chr6	25154795	C	G	GGGAGCACCACCTAGTGATGTCTGAG	TTATGGTGCAGAAGCACAGAATCGG
HdrR	chr13	12608185	C	G	ACGTTGATTTTGCACTGACGTTTGC	CTAGTAGGGAGGCTTCGTAAGGTGC
HdrR	chr7	17776985	T	C	ATTTTGGCTGGGGGACCTGAAACAG	ACAAGAGGCCACTAAAGTCCCACTCC
HdrR	chr18	12893252	A	G	TCCAGCCATAGGTGAAATCTACAAAGC	GCCAAGCTGTTCAATGGTTTAAGGC
HdrR	chr6	7190804	T	C	GTTTTTGCCTTTTGTGGTGCAGGGC	AGTCATAAGGGCGTTTCAAAAGCTGGC
HdrR	chr1	23489939	C	T	CTTTCCTGTTCTCACTCACAGGCCC	ATCCAGGTGTGCGTATGGCTGTTTC
HdrR	chr21	27091378	T	C	TGGTAGGCTGTTCCAGAGGTCAAAGG	TGGGTCAAAATCTCTCAAGGCGCTC
HdrR	chr16	5095846	A	G	ATGAGTCTTAGTTGCCATGGGCGGG	ACGGCATAAGCACACACACAGAGAC
HdrR	chr7	3668467	T	C	TGTGTTGACTTTGTCTGTGCTTTGC	TTCAAGCACACCGTCCCGTTCTGTC

Table S6 Details of sequence coverage for the additional inbred medaka lines. The table provides percent read alignment for each of the libraries sequenced for each inbred strain. Total coverage of the genome and median read depth is given for the total sequence for all libraries from that strain.

**Strain**	**HNI**	**HSOK**	**Kaga**	**Nilan**
**Aligned reads by library type (%)**				
3kb mate-pair 100bp reads	50	59,7	56,2	56.2
54bp reads	69,3	66	60,9	60.9
300bp paired-end	82,5	81	77,3	77.3
500bp paired-end	82,4	81	76,5	76.5
**Coverage of reference genome (%)**	78,4	77,8	78,4	76,9
**Median coverage at locations covered at least once**	113	108	119,0	125
