# Supplementary material for: Genomic and Phenotypic Characterization of a Wild Medaka Population: Towards the Establishment of an Isogenic Population Genetic Resource in Fish
Source: G3 (Bethesda). 2014 Jan 9;4(3):433–45. doi: 10.1534/g3.113.008722 (PMC3962483; doi:10.1534/g3.113.008722)
Supplement: Supporting Information [file supp_g3.113.008722_FigureS1.pdf]

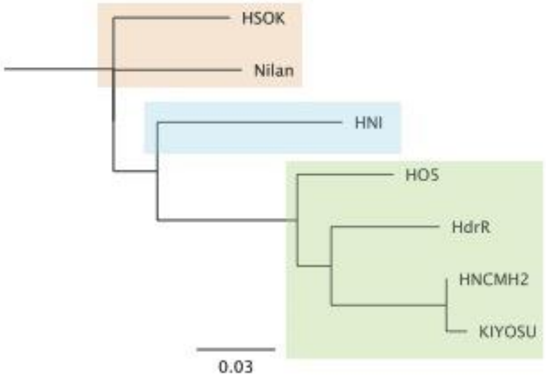

Figure S1 Boxplots of measured morhpometric features in the different inbred strains. A) Dorsal features. B) Lateral features. X-axis: Pixel values for each measurement. Grey shading: Southern inbred strains. Yellow shading: Northern inbred strains.
