# Supplementary material for: Genomic and Phenotypic Characterization of a Wild Medaka Population: Towards the Establishment of an Isogenic Population Genetic Resource in Fish
Source: G3 (Bethesda). 2014 Jan 9;4(3):433–45. doi: 10.1534/g3.113.008722 (PMC3962483; doi:10.1534/g3.113.008722)
Supplement: Supporting Information [file supp_g3.113.008722_FigureS2.pdf]

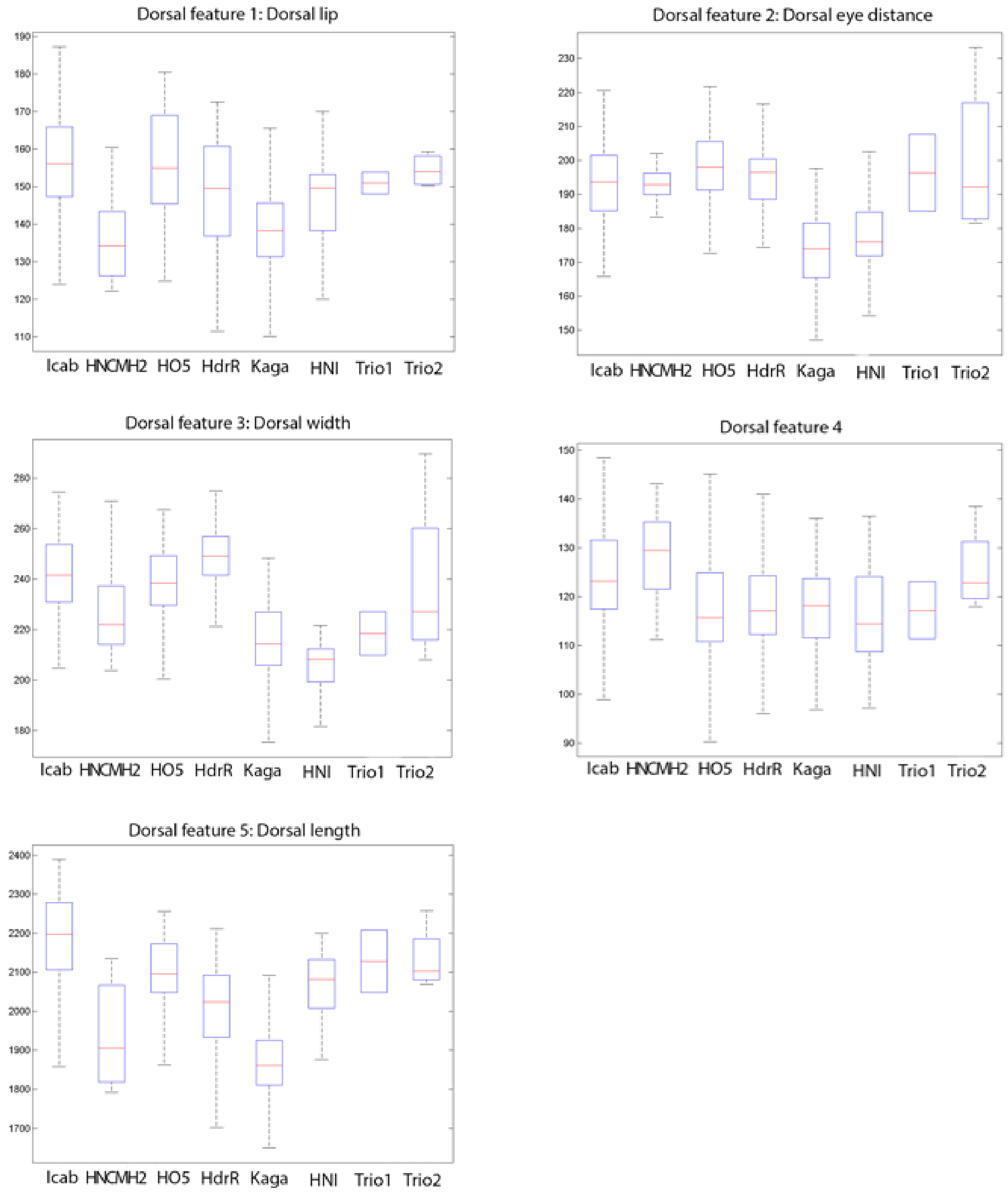

Figure S2 Morphometric analysis of four inbred Southern lines, two inbred Northern lines and two trios from the Kiyosu wild population: Dorsal features. The respective definitions of Features 1 to 5 are indicated in Figure 6A. A significant difference in all features between the six inbred lines is observed. The Kiyosu morphometric analysis is preliminary as the dataset has low sample numbers (N < 10), whereas for the inbred lines N > 75. The differences between the Kiyosu datasets to the other datasets are therefore not statistically significant.
