# Supplementary material for: Genomic and Phenotypic Characterization of a Wild Medaka Population: Towards the Establishment of an Isogenic Population Genetic Resource in Fish
Source: G3 (Bethesda). 2014 Jan 9;4(3):433–45. doi: 10.1534/g3.113.008722 (PMC3962483; doi:10.1534/g3.113.008722)
Supplement: Supporting Information [file supp_g3.113.008722_TableS1.pdf]

Table S1 Microsatellite markers used. Oligonucletoide primer sequences, genomic locations and amplicon lengths for the microsatellite markers analyzed in the Kiyosu population sample.

Linkage Group	Forward Primer	Reverse Primer	Genomic location	Estimated amplicon size
LG1	AGCATTTCTAGTTTGCGTTT	CTAGTTTTTCCGGACAGTCA	1:24815630:24815850:1	202
LG2	ATATCGACAGGTATGGATGG	TCACATCTAGACCTGGAAGC	2:27141500:27141900:1	243
LG3	TTATGATCCCCATATTTGATT	AGAGGGACACTGGGACAG	3:9396729:9397030:1	301
LG4	GCTTTAAATTAGAGTTCATAATACTGC	GCTTTCATTAACATCAGGAAG	4:22460213:22460453:1	229
LG5	GTTTTTGAAAGTTTTATTTTTGG	TCAAAGCTAGTATTATGTAAACACAG	5:8443091:8443291:1	181
LG6	ATGTCCACCGACAAACAC	AGTAGATGCCCAGTGAGAGA	6:10841500:10841800:1	250
LG7	AGCTTGCCCTTTTTCTACAT	GTTTGGAGACCCACTGTTT	7:19386200:19386460:1	188
LG8	GCCAAAATGCCTTTTTATTT	TCCACATCAGAGCACATTC	8:21636852:21637100:1	209
LG9	TTTTCATCTGCTCCTGATTT	AGAGTTTCAGCAAGCAATGT	9:26192300:26192600:1	212
LG10	CACCAGCTGAGTTTGTAATG	CAAGAGAAGGGAAGTCGTG	10:10113640:10113860:1	208
LG11	ACAAGTATAGCTGCCCTCAG	CCTGCTTTCTTTTTGATTTT	11:4573925:4574200:1	205
LG12	GCTGTTCACAGCTGATGTC	ATATTGGCACAAGCTGTTTT	12:15240800:15241200:1	202
LG13	TTAGGCTCAATCTGTTATTTACAGATTAC	ATCCACAGGCTCAGCATCTT	13:10169674:10169904:1	231
LG14	TTGCTGTCCCTGTGTGTTTG	GCTTGAAGGCAACAGCTTTG	14:7011652:7011851:1	200
LG15	TGCCATAAATGAGGGAATTTAC	TCAGTGTGAATCAGTTTCCTGT	15:7515398:7515597:1	200
LG16	AATGACTTGGTGTCATATAATTCCA	AATTAAATATCATCTTGGCAACATTATA	16:7247259:7247458:1	200
LG17	GGATTGCTGCTGAGTGTAATTC	CAATTTCCAGTTTCACTGACTGG	17:8333779:8333969:1	191
LG18	AGCTTAGCAGACCGTTTTAGG	CCTGCCGTGGATCTCAG	18:5214059:5214245:1	187
LG19	GGTTTTTGCATCCTGCC	GCTACCGAGTCCTTTGAATAAAG	19:13774098:13774297:1	200
LG20	AAAAGCTGTATGAACAGATTTGC	TGAGAGCCTGTTGCCTTC	20:7665987:7666186:1	200
LG21	TAGCGGAGGCTGGCTG	CAGAGCGCGCGGAGC	21:10273689:10273888:1	200
LG22	AGAAAAGGAATGAGCCAGACC	GTGTTTGTGCCTGGAAATAGG	22:7909633:7909831:1	199
LG23	GTGGAGGCTGCCGACGG	CTCTGCTGTCTGCCTGCC	23:8615246:8615443:1	198
LG24	AGTTTTTGGCTACATTCATAGTCAG	GGAGAGGGGTTTCTAGGGA	24:9528803:9529032:1	230
