# Supplementary material for: Genomic and Phenotypic Characterization of a Wild Medaka Population: Towards the Establishment of an Isogenic Population Genetic Resource in Fish
Source: G3 (Bethesda). 2014 Jan 9;4(3):433–45. doi: 10.1534/g3.113.008722 (PMC3962483; doi:10.1534/g3.113.008722)
Supplement: Supporting Information [file supp_g3.113.008722_TableS2.pdf]

**Table S2 Microsatellite alleles detected in wild Kiyosu Population.** Microsatellite alleles were tested in 109 fish from the wild Kiyosu Population. PCR amplification failed in 4 fish (samples 1, 5, 6, 7) giving allele results for 105 fish. For each microsatellite assay labeled as described in Supplementary Table 1, the two alleles identified in each assayed Kiyosu individual are listed.

| Sample | D-loop Sequencing | LG3 | LG3 | LG6 | LG6 | LG13 | LG13 | LG14 | LG14 | LG16 | LG16 | LG17 | LG17 | LG18 | LG18 | LG22 | LG22 | LG24 | LG24 |
|---|---|---|---|---|---|---|---|---|---|---|---|---|---|---|---|---|---|---|---|
| 1 | C | - | - | - | - | - | - | - | - | - | - | - | - | - | - | - | - | - | - |
| 2 | G | L | M | C | H | A | A | A | A | A | A | C | C | B | B | K | K | A | B |
| 3 | H | E | E | F | I | D | A | F | K | A | A | C | C | B | B | O | O | A | E |
| 4 | H | U | U | - | - | G | A | A | B | A | A | C | F | B | B | A | A | B | D |
| 5 | A | - | - | - | - | - | - | - | - | - | - | - | - | - | - | - | - | - | - |
| 6 | H | - | - | - | - | - | - | - | - | - | - | - | - | - | - | - | - | - | - |
| 7 | H | - | - | - | - | - | - | - | - | - | - | - | - | - | - | - | - | - | - |
| 8 | C | Q | S | A | E | G | G | A | A | A | A | A | A | A | B | E | H | B | C |
| 9 | H | N | N | - | - | - | - | - | - | A | A | A | A | - | - | - | - | - | - |
| 10 | C | B | O | - | - | - | - | - | - | A | B | F | F | - | - | - | - | - | - |
| 11 | H | M | Q | - | - | - | - | - | - | A | A | B | C | - | - | - | - | - | - |
| 12 | C | E | L | - | - | - | - | - | - | A | A | C | G | - | - | - | - | - | - |
| 13 | H | F | M | A | E | A | F | A | N | A | A | B | D | B | B | F | N | A | C |
| 14 | G | B | L | A | G | A | A | A | K | A | A | A | D | B | B | D | H | A | A |
| 15 | A | C | J | E | F | D | E | A | G | A | A | C | F | A | B | B | E | A | D |
| 16 | H | H | M | A | A | J | K | D | L | A | A | B | C | B | B | E | I | C | K |
| 17 | H | M | M | D | E | A | A | B | C | A | A | C | E | B | B | E | E | B | C |
| 18 | E | A | L | A | E | A | D | B | B | A | A | B | E | B | B | J | J | A | D |
| 19 | D | E | I | F | G | A | D | O | P | A | A | C | D | B | B | B | C | B | D |
| 20 | H | F | K | A | E | A | I | A | M | A | A | C | E | B | C | E | E | C | E |
| 21 | H | Q | Q | A | A | A | B | G | G | A | A | C | F | A | B | A | J | D | G |
| 22 | C | K | S | E | I | D | I | A | J | A | A | D | F | A | B | E | J | D | D |
| 23 | C | M | M | A | I | D | H | A | G | A | A | A | A | B | B | G | G | C | E |
| 24 | H | K | N | D | I | A | C | C | D | A | A | B | C | B | B | B | C | A | C |
| 25 | H | A | J | E | F | A | A | C | H | A | A | D | F | B | B | C | H | A | C |
| 26 | C | U | U | D | E | A | E | A | A | A | A | A | D | B | B | C | E | C | E |
| 27 | - | G | I | E | G | A | C | B | C | A | A | A | E | B | B | F | M | A | L |
| 28 | C | H | O | B | E | D | F | B | B | A | A | C | D | B | B | A | A | A | C |

| | | | | | | | | | | | | | | | | | | | |
|---|---|---|---|---|---|---|---|---|---|---|---|---|---|---|---|---|---|---|---|
| 29 | C | F | I | D | E | A | A | - | - | A | A | B | E | B | B | E | L | - | - |
| 30 | - | A | I | A | E | D | D | A | A | A | A | A | D | B | B | C | C | A | D |
| 31 | G | K | R | A | H | - | - | - | - | A | A | B | D | B | B | G | I | - | - |
| 32 | C | B | P | E | G | A | A | A | C | A | A | B | C | B | C | A | E | A | B |
| 33 | - | I | K | A | H | B | B | D | D | A | A | C | E | A | B | C | J | F | F |
| 34 | G | E | E | A | G | A | E | I | O | A | A | B | C | A | B | I | I | B | B |
| 35 | B | D | L | A | G | D | D | B | I | A | A | A | D | B | B | A | E | B | C |
| 36 | G | P | P | B | F | A | B | B | C | A | A | C | D | B | B | C | G | A | D |
| 37 | C | K | Q | E | G | B | D | A | M | A | A | B | D | A | B | C | N | A | E |
| 38 | C | D | G | A | G | A | C | B | G | A | A | C | D | B | B | E | E | A | A |
| 39 | C | E | J | A | C | D | F | C | J | A | A | A | F | A | B | C | H | C | E |
| 40 | H | L | O | A | F | C | D | D | F | A | A | B | D | A | B | C | E | A | C |
| 41 | G | E | O | A | A | E | H | A | B | A | A | B | D | B | B | K | K | A | E |
| 42 | C | D | J | E | G | A | C | B | M | A | B | C | H | A | B | E | M | B | C |
| 43 | H | R | R | C | G | B | B | I | O | C | D | C | C | A | B | E | E | F | J |
| 44 | H | C | P | F | I | A | H | D | K | A | A | D | D | A | B | E | P | E | E |
| 45 | H | - | - | F | G | B | B | B | B | A | B | A | G | A | B | C | E | F | F |
| 46 | C | A | S | A | I | A | C | A | A | A | A | A | G | B | B | N | N | C | C |
| 47 | C | L | M | E | G | C | G | C | H | A | A | A | E | B | B | A | E | C | C |
| 48 | H | E | R | A | E | A | D | B | F | A | A | C | D | B | B | C | G | A | D |
| 49 | H | I | L | - | - | - | - | - | - | A | A | B | C | - | - | - | - | - | - |
| 50 | C | - | - | - | - | - | - | - | - | A | A | B | B | - | - | - | - | - | - |
| 51 | E | F | G | B | E | A | D | A | M | A | A | A | A | A | B | A | H | A | D |
| 52 | H | G | L | E | E | A | C | F | F | A | A | A | J | B | B | C | C | A | A |
| 53 | H | A | G | A | D | F | I | B | I | A | A | C | E | B | B | H | I | A | D |
| 54 | C | B | K | F | F | E | F | A | A | A | A | C | C | B | B | E | O | A | E |
| 55 | H | O | Q | A | E | A | D | F | I | A | A | C | D | B | B | E | O | A | C |
| 56 | H | E | Q | F | J | A | A | G | I | A | A | B | B | B | B | F | L | B | C |
| 57 | H | M | P | A | E | E | G | E | J | A | A | B | C | B | B | C | G | A | C |
| 58 | C | A | L | A | A | C | H | A | B | A | A | A | A | B | B | B | E | C | E |
| 59 | H | L | O | D | G | D | E | N | N | A | A | A | A | B | B | C | O | A | C |
| 60 | B | H | O | A | A | A | C | H | N | A | A | A | F | B | B | D | L | A | B |

| | | | | | | | | | | | | | | | | | | | |
|---|---|---|---|---|---|---|---|---|---|---|---|---|---|---|---|---|---|---|---|
| 61 | H | M | S | A | H | D | D | C | J | A | A | C | F | B | B | B | O | B | D |
| 62 | C | H | Q | G | I | B | B | M | M | A | A | A | A | B | B | B | J | F | I |
| 63 | D | K | S | A | F | A | D | K | M | A | A | B | B | B | B | E | E | A | G |
| 64 | H | C | D | A | D | B | F | D | N | A | A | B | C | B | B | B | N | B | C |
| 65 | H | F | K | A | A | B | C | A | C | A | A | A | A | B | B | A | A | C | D |
| 66 | H | E | H | A | E | A | A | M | N | A | A | A | D | B | B | C | L | A | C |
| 67 | H | E | Q | A | E | A | D | A | L | A | A | B | B | A | B | B | B | A | C |
| 68 | H | K | O | D | E | A | A | A | I | A | A | E | E | B | B | E | I | E | E |
| 69 | H | E | F | E | G | A | C | A | B | A | A | B | B | B | B | N | P | B | F |
| 70 | C | P | S | A | G | C | D | B | K | A | A | B | B | B | B | D | J | B | C |
| 71 | H | K | N | E | G | C | D | A | B | A | A | A | J | B | B | G | L | C | D |
| 72 | C | E | M | C | G | C | D | G | N | A | A | A | F | B | B | - | - | A | C |
| 73 | G | K | P | G | G | D | H | C | O | A | A | A | A | B | B | B | E | A | B |
| 74 | H | D | K | A | A | A | D | B | B | A | A | C | F | B | B | C | M | A | B |
| 75 | D | E | H | E | G | A | C | A | C | A | A | B | B | B | B | C | K | C | C |
| 76 | C | A | L | A | F | A | C | A | C | A | A | C | E | B | B | B | C | B | D |
| 77 | C | N | Q | A | A | A | F | A | A | A | A | A | D | B | B | A | A | A | C |
| 78 | H | E | M | A | I | A | C | A | A | A | A | A | D | A | B | F | I | A | D |
| 79 | H | B | T | - | - | D | H | I | J | A | A | C | C | - | - | - | - | B | C |
| 80 | H | A | E | G | H | A | C | F | J | A | A | C | D | B | B | C | N | B | B |
| 81 | D | F | L | A | J | C | C | E | H | A | A | B | C | B | B | B | E | A | C |
| 82 | H | M | M | A | I | A | C | B | P | A | A | B | D | B | B | E | E | B | C |
| 83 | H | E | K | A | F | A | H | A | O | A | A | A | D | A | B | B | L | B | B |
| 84 | D | E | M | A | E | A | A | A | A | A | A | A | D | B | B | B | E | C | C |
| 85 | C | A | G | E | E | A | A | H | I | A | A | B | C | A | B | J | O | A | D |
| 86 | G | E | M | A | E | D | D | B | I | A | A | A | A | B | B | C | C | B | D |
| 87 | H | M | P | D | E | C | D | A | A | A | A | B | C | A | B | L | L | A | D |
| 88 | H | E | L | A | G | A | E | A | B | A | A | C | F | B | B | B | M | A | C |
| 89 | C | E | E | B | E | A | G | D | G | A | A | C | D | B | B | E | G | A | C |
| 90 | H | B | K | A | A | D | F | G | I | A | A | F | F | A | B | J | L | A | C |
| 91 | H | A | I | A | A | A | C | A | N | A | A | B | B | A | B | C | C | C | D |
| 92 | C | F | P | A | G | F | I | A | D | A | A | B | G | A | B | G | I | A | C |

| | | | | | | | | | | | | | | | | | | | |
|---|---|---|---|---|---|---|---|---|---|---|---|---|---|---|---|---|---|---|---|
| 93 | H | I | N | F | J | A | H | A | G | A | A | C | I | A | B | C | C | A | A |
| 94 | G | E | K | E | H | - | - | - | - | A | A | B | I | A | B | - | - | - | - |
| 95 | H | G | O | E | E | A | B | A | A | A | A | A | A | B | B | C | C | D | D |
| 96 | H | F | P | A | C | A | E | I | G | A | A | A | A | A | B | K | L | B | D |
| 97 | H | A | M | C | F | B | D | A | C | A | A | A | I | B | B | G | H | C | D |
| 98 | D | F | L | E | E | D | D | A | M | A | A | A | A | B | B | B | C | A | C |
| 99 | H | G | I | E | I | A | A | G | H | A | A | A | F | B | D | K | K | A | B |
| 100 | H | B | P | A | G | A | C | A | I | A | A | B | C | B | B | B | E | A | D |
| 101 | H | E | E | A | C | D | D | A | I | A | A | A | A | A | B | C | D | A | D |
| 102 | F | E | G | E | H | A | D | B | F | A | A | A | F | A | B | E | G | D | E |
| 103 | C | I | T | A | E | C | E | D | J | A | A | B | E | B | C | C | K | A | C |
| 104 | H | D | S | A | F | F | H | A | A | A | A | B | B | A | B | E | K | C | D |
| 105 | H | L | M | C | E | C | D | D | F | A | A | C | D | B | B | C | C | D | E |
| 106 | C | F | O | A | G | A | D | I | O | A | A | C | D | B | B | G | G | B | C |
| 107 | H | N | O | G | G | A | I | B | C | A | A | B | C | A | B | J | L | A | C |
| 108 | H | E | K | A | E | A | C | H | L | A | A | A | A | A | B | C | E | A | E |
| 109 | H | E | I | A | I | A | C | D | D | A | A | B | C | A | B | E | N | A | A |
