# Supplementary material for: Genomic and Phenotypic Characterization of a Wild Medaka Population: Towards the Establishment of an Isogenic Population Genetic Resource in Fish
Source: G3 (Bethesda). 2014 Jan 9;4(3):433–45. doi: 10.1534/g3.113.008722 (PMC3962483; doi:10.1534/g3.113.008722)
Supplement: Supporting Information [file supp_g3.113.008722_TableS3.pdf]

**Table S3 Introgression analysis.** Tests for introgression of the P3 strain into P1 or P2 strains that are more closely related with each other than to P1, using stickleback as the outgroup (O). D% is the introgression test statistic, on which the standard error (SE), 95% confidence intervals (CI) and Z-scores were estimated using block-wise jackknife (Durand *et al.* 2011).

| P1 | P2 | P3 | O | D% | SE | 95% CI | Z-score |
|---|---|---|---|---|---|---|---|
| **Kaga** | **Hni** | **HdrR** | Stickleback | **3.6** | **1.3** | **2.5** | **2.77** |
| Kaga | Hni | Wild Southern | " | 2.6 | 1 | 2 | 2.5 |
| Kaga | Hni | Wild Southern (SNPs with HdrR) | " | -4 | 3.6 | 7.1 | 1.1 |
| **Nilan** | **Hsok** | **HdrR** | **"** | **–12.3** | **4.66** | **9.1** | **2.64** |
| Nilan | Hsok | Wild Southern | " | –10.4 | 5.27 | 10.3 | 1.96 |
| Nilan | Hsok | Wild Southern (SNPs with HdrR) | " | 7.6 | 6.5 | 12.8 | 0.6 |
| Nilan | Hsok | Kaga | " | –0.7 | 2.5 | 4.9 | 0.28 |

Tests for introgression of the P3 strain into P1 or P2 strains that are more closely related with each other than to P1, using stickleback as the outgroup (O). D% is the introgression test statistic, on which the standard error (SE), 95% confidence intervals (CI) and Z-scores were estimated using block-wise jackknife.
