# Supplementary material for: Genomic and Phenotypic Characterization of a Wild Medaka Population: Towards the Establishment of an Isogenic Population Genetic Resource in Fish
Source: G3 (Bethesda). 2014 Jan 9;4(3):433–45. doi: 10.1534/g3.113.008722 (PMC3962483; doi:10.1534/g3.113.008722)
Supplement: Supporting Information [file supp_g3.113.008722_TableS4.pdf]

Table S4 iHS analysis. Numbers of SNPs showing evidence of recent positive selection in regions with different levels of evolutionary conservation versus those expected at random. Expected values, their standard deviations and p-values were estimated from permutation tests.

Type	Observed	Expected	Standard deviation (+/-)	Fold change	Direction of difference	P
Non-conserved	87	60	7	1.45	Enriched	<0.005
At least 1 other teleost	105	131	7	0.80	Depleted	<0.005
At least 2 other teleosts	95	110	7	0.86	"	0.014
At least 3 other teleosts	72	89	7	0.81	"	0.008
Four other teleosts	38	27	5	1.41	Enriched	0.032

Numbers of SNPs showing evidence of recent positive selection in regions with different levels of evolutionary conservation versus those expected at random. Expected values, their standard deviations and p-values were estimated from permutation tests.
