# Supplementary material for: Genomic and Phenotypic Characterization of a Wild Medaka Population: Towards the Establishment of an Isogenic Population Genetic Resource in Fish
Source: G3 (Bethesda). 2014 Jan 9;4(3):433–45. doi: 10.1534/g3.113.008722 (PMC3962483; doi:10.1534/g3.113.008722)
Supplement: Supporting Information [file supp_g3.113.008722_TableS5.pdf]

Table S5 Primers designed for SNP verification sequencing. PCR amplification primers are given for the SNP resequencing amplicons. For the coding SNPs, the original reference sequence indicated a coding triplet, whereas a stop codon was indicated by our height throughput sequencing. The status column indicates whether PCR amplification and sequencing confirmed the new sequence.

NONSENSE SNPs

Strain	Chromosome	Position	Reference triplet	Detected triplet	Reference amino acid	Forward primer	Reverse primer
HNI	chr23	20491840	TTA	TAA	L	TCAGTGGCTGTAAAAGGCTGTAGATTG	ACCACACAGATGCGAAAAACACATCC
HNI	chr8	1955292	CGA	TGA	R	ACCAGAATCCTTCTCCTCCACCTGC	CAAGCTGTGTAAGTGGGCCTGAGTC
HNI	chr7	1468063	TGG	TAG	W	AGTGTCCCATTTTGCCTTCAACAGC	CGGTTGCTTCTGCAAAGCCTATTCTCC
HNI	chr18	2964751	AAG	TAG	K	CTTTGCGTAGAATCTGATGGGACAGC	TGTATGCCTGCCTTTTCAATCAGCG
HNI	chr2	20573966	CAG	TAG	Q	CCGAGGTAAATTCTTTTCTGGTCAGTGG	GGGTTGTTTGTGCTTGGCTTCCCTG
HNI	chr14	15525786	TAC	TAA	Y	ACAACGACGTGAATATTCTGGAGGGG	AAGTCGCTTTTTACTGCGCACCTGG
Kaga	chr3	2245314	TCA	TGA	S	TCTTGCAGCTGGGGGAGGTGATGG	AGTTCCTCGTCTGCAATTGGTTGGC
Kaga	chr5	14257733	CAA	TAA	Q	TCAATTATTATCGTGGGATGTACTGGTGC	TGTGGATGGATGGAACAAGCTCAACG
Kaga	chr14	7969562	CAA	TAA	Q	AGCTCAAGGTGGTGTAGGTTCCAGG	CTCGTGGGTCTCCCGTTCTCTCATC
Kaga	chr13	5610005	TCG	TAG	S	CGCCAGATCAAAGATAGGGGGAAAGC	TATGACGTTCACACTGGAGAAGGGGC
Nilan	chr8	22537356	CGA	TGA	R	CCACAGAGCCACAGGAGATGTGATG	AGAATCCTAAAAGCAGCAGACGCCG
Nilan	chr22	1757977	GGA	TGA	G	TCGTTTCCTGCAGATCCTGAGGTGG	GGGTCAGGGTTTTAGCCGTTTCTGC
Nilan	chr16	6784490	TGT	TGA	C	TCACTTTTGCGTCTCTGTCTTTAGGTGG	TGTCTGACAGCTTAACAAGGATCTGTTTG
Nilan	chr7	4911752	TGG	TAG	W	TCTGCTGTACGCAGCGGACTTTTTC	AACTTTAAGCTTTTCCTGGCGCAGC
Nilan	chr18	17668284	AAA	TAA	K	ACACACCTTTCTTGACTTCAGCCGC	AACGAGAAGACAGAAGACGGTGAGGC
Nilan	chr15	11785459	GAG	TAG	E	ACATTTCTGACTAATTTCAGCAGCAGGTC	CGTAGTTGGACTTTATGGAAGCCAGG

NON-CODING SNPs (Kaga)

Strain	Chromosome	Position	Reference base	Detected base	Forward primer	Reverse primer
Kaga	chr3	8401434	T	G	AGGTTTGTTTACCAGTGATTGTGACTGAG	GGTGCGTTATGTAGCTGCTGTTCGG
Kaga	chr4	31533375	G	T	AGCAGCCAGTGCCAAGAAAACAGAG	TTTCAATGGCCCTTTAGGGGCTCCG
Kaga	chr6	15965403	G	C	TGCACGATGTTGCAAAATATTACCCCC	CGTCTTGGTCACAGCGAACTCTAACG

Kaga	chr7	27519929	G	A	TGCACTCTTGCCATAAACAGTCATCC	TGTCTGTCTGGAAGCAGCATAACCC
Kaga	chr19	19825516	A	T	CCTGCACAAAACCCACACATACACG	AGGCAGAGTTTGACGTGGATCAAGC
Kaga	chr22	5161675	G	A	GACCTTTTAACTGGGGCCCTGTGTG	TCTCCTCCCCCTTTGTTGTGTGCTG
Kaga	chr20	9670880	A	T	GCAAGCTGCCCTAACCAATGATCCG	CTCACATGCCTAGCAGCAGTGACAG
Kaga	chr22	24304734	T	C	TGGGGTTCTAGTGAAGGAAGGCTTGG	GCCTCTTTTAGCCGTCCTCCTGTTG
Kaga	chr5	28448196	A	T	CTGATGTGACACACTCCAAAAACAGGC	ACGAGCCTGTTGGGAGTCAAAAAGC
Kaga	chr17	2618509	C	G	GCTGCTTCTAAGGTCAGTCCGTTTG	GACAACTAAAAGCAGCAAGCGCAGC

RANDOM SNPs (HdrR)

Strain	Chromosome	Position	Reference base	Detected base	Forward primer	Reverse primer
HdrR	chr1	16324653	C	A	CAAGGGAGGCACCAATGAACCTGAG	TTTTGATGTGGAGGAACAGTAGGCTG
HdrR	chr1	33512684	T	G	TGTTTGTTGCAGAGCGAAGCAGCAG	GACCGTGAATGTAAAAGGGAGCCCG
HdrR	chr9	31027771	T	G	GCACCAGGCTTGTCCACAGTTAAGG	AAGACATCCGTGTCCGTTCCAGTGC
HdrR	chr16	3550201	C	A	AGCCGACGTGGCCTTTGTGTGGG	TCTTCTGGTGGTTTCTACCCTCTGGG
HdrR	chr1	27318612	A	G	CAAGCAGGTTTTGCTTTCAACTCCGC	ACCTGCCAAAAGTGAAACCTAACGGC
HdrR	chr16	5495844	A	G	GCAACCATGGCAGTTGCACCATAAAC	TGAGCGCTCTTCCTCTCATCCATGC
HdrR	chr1	23401471	C	T	CCGATACGCTGGTTTCTACGGTCTG	TGTCTGTGTTTGAACTGTGTCTCCAGC
HdrR	chr1	15819568	A	G	AAAGAGTCAGATGGAAGAGCTGAAAGATG	TGATTGCGTGTACTCCTCCTCCAAG
HdrR	chr1	19723612	C	T	ACATGGTACTCACAACGCTCATTGGC	CACAGAAGTACCGCTGAAGGTGCTG
HdrR	chr22	21769957	T	C	GTGAGAGCGCTGGAGATGAGAGGAG	CTGGAAAACACATTTCACGCTGCGG
HdrR	chr8	2893859	A	G	ACAAGTTGGGCATATCCACAGGACG	GTTTACCACACCGATAATTTTAAC
HdrR	chr16	20019740	T	C	GGGACGCGAAGGTGAGCAATGAATC	TCTGGCAGAGACCCACCAGCTTAC
HdrR	chr8	14657015	C	T	CATGCCTGTGTGCTTGACTATTGCAG	CCGGTTCAAGAAGTCGTTAAACTGG
HdrR	chr13	5993620	T	A	CGTTTTTTAAGGAAAACCTTGGATGG	TGGACTGAGTGACCCCTAACCCCTC
HdrR	chr9	11937557	T	C	TCCTAACAGCAAAGGACTCAAAACCC	GTGACACAGGGCTGTGCATAGAGAG
HdrR	chr24	17741133	G	T	GTCCACCTTTTTCAGAGCTCTTTCTGAC	TCAGGCCTTCTTTGTCACTGCCTGG
HdrR	chr2	15141629	G	A	ATGTCCCCCTAAAATCCCTCTAGGC	CATTGCTGCAGTCCGGAGTCAAAGG
HdrR	chr22	21202891	A	G	TAGGTGTGAGTGCGGGAGTGAATGG	TCCAGGTCCAGGTCCAGGTCTATCC
HdrR	chr12	5608651	A	G	CTGCTGCATTTAAACATCTGACTTTCAGG	TACATGCAGGCAAACACAATGAAAATGTC
HdrR	chr9	15611521	C	A	GCATGTGTGACCCTGTCCGTGTAAG	GCTTATCAACATGCCAATGTCCCCC

HdrR	chr22	21589634	A	G	AAATGCAGGGTTGAACGCTCTAATGC	TCCAGGATTTCCAGGATTTTTGCCTC
HdrR	chr1	21776066	G	T	GAACCTGATCAGGAAGAAGTTAGGG	TGCCTGTTCTTTGAAACTGTGCCTC
HdrR	chr1	21103193	A	T	TCCAGCCCGCTCAGATGTTGTAGAC	GAATGGGGAGTCTGTATCGCCCCTG
HdrR	chr1	33292558	A	C	ACGGGCACGTGACTGGTGTCTATG	GGATACCGTGTGACGGTGCGTAATAG
HdrR	chr10	22338538	C	A	AACCCCAACAACCCTTTTGGAGTGC	TGAGGCGACTCAGCCTTACAGCTTG
HdrR	chr1	29019329	G	C	CGCCTTGGTCTGCATCAAGAGTGAC	TGCGCTCCTAAGCTTTCTCTCAGCG
HdrR	chr1	28914573	C	G	AGCAGGCACTGTGAAGAATACCACG	ACTTCAAGGCTCACTTTTTGGTGCC
HdrR	chr13	21682092	A	G	TGTTTTACCCTTAAACAATGGTTTGCTGC	ATGGAAATCTGCACAGGTGCAAGCC
HdrR	chr15	27757265	A	C	CATCAAAACCTAAGACAACCTCCTGCC	TGTGACGTTTTTGTGTTTCTGTGAGAACG
HdrR	chr20	3264988	C	T	GAGGTGTGAACTGTCGTCTGAAGGC	AGGCAGTTTTATGTTTGAGCGCCCC
HdrR	chr1	22961788	T	A	GCAGCCACTGGTATTTTGTGGGGAC	CCTGGGGGACGTTCCCTCGATTAG
HdrR	chr6	25154795	C	G	GGGAGCACCACCTAGTGATGTCTGAG	TTATGGTGCAGAAGCACAGAATCGG
HdrR	chr13	12608185	C	G	ACGTTGATTTTGCACTGACGTTTGC	CTAGTAGGGAGGCTTCGTAAGGTGC
HdrR	chr7	17776985	T	C	ATTTTGGCTGGGGGACCTGAAACAG	ACAAGAGGCCACTAAAGTCCCACTCC
HdrR	chr18	12893252	A	G	TCCAGCCATAGGTGAAATCTACAAAGC	GCCAAGCTGTTCAATGGTTTAAGGC
HdrR	chr6	7190804	T	C	GTTTTTGCCTTTTGTGGTGCAGGGC	AGTCATAAGGGCGTTTCAAAAGCTGGC
HdrR	chr1	23489939	C	T	CTTTCCTGTTCTCACTCACAGGCCC	ATCCAGGTGTGCGTATGGCTGTTTC
HdrR	chr21	27091378	T	C	TGGTAGGCTGTTCCAGAGGTCAAAGG	TGGGTCAAAATCTCTCAAGGCGCTC
HdrR	chr16	5095846	A	G	ATGAGTCTTAGTTGCCATGGGCGGG	ACGGCATAAGCACACACACAGAGAC
HdrR	chr7	3668467	T	C	TGTGTTGACTTTGTCTGTGCTTTGC	TTCAAGCACACCGTCCCGTTCTGTC
