# Supplementary material for: Genomic and Phenotypic Characterization of a Wild Medaka Population: Towards the Establishment of an Isogenic Population Genetic Resource in Fish
Source: G3 (Bethesda). 2014 Jan 9;4(3):433–45. doi: 10.1534/g3.113.008722 (PMC3962483; doi:10.1534/g3.113.008722)
Supplement: Supporting Information [file supp_g3.113.008722_TableS6.pdf]

Table S6 Details of sequence coverage for the additional inbred medaka lines. The table provides percent read alignment for each of the libraries sequenced for each inbred strain. Total coverage of the genome and median read depth is given for the total sequence for all libraries from that strain.

Strain	HNI	HSOK	Kaga	Nilan
**Aligned reads by library type (%)**				
3kb mate-pair 100bp reads	50	59,7	56,2	56.2
54bp reads	69,3	66	60,9	60.9
300bp paired-end	82,5	81	77,3	77.3
500bp paired-end	82,4	81	76,5	76.5
**Coverage of reference genome (%)**	78,4	77,8	78,4	76,9
**Median coverage at locations covered at least once**	113	108	119,0	125
